# Resonance-Assisted Hydrogen Bond—Revisiting the Original Concept in the Context of Its Criticism in the Literature

**DOI:** 10.3390/ijms23010233

**Published:** 2021-12-26

**Authors:** Małgorzata Domagała, Sílvia Simon, Marcin Palusiak

**Affiliations:** 1 Department of Physical Chemistry, Faculty of Chemistry, University of Łódź, Pomorska 163/165, 90236 Łódź, Poland; marcin.palusiak@chemia.uni.lodz.pl; 2 Institut de Química Computacional I Catàlisi, Departament de Química, Universitat de Girona, C/Ma Aurèlia Capmany, 69, 17003 Girona, Spain

**Keywords:** RAHB, many-body approach, multicentre delocalization indices

## Abstract

In the presented research, we address the original concept of resonance-assisted hydrogen bonding (RAHB) by means of the many-body interaction approach and electron density delocalization analysis. The investigated molecular patterns of RAHBs are open chains consisting of two to six molecules in which the intermolecular hydrogen bond stabilizes the complex. Non-RAHB counterparts are considered to be reference systems. The results show the influence of the neighbour monomers on the unsaturated chains in terms of the many-body interaction energy contribution. Exploring the relation between the energy parameters and the growing number of molecules in the chain, we give an explicit extrapolation of the interaction energy and its components in the infinite chain. Electron delocalization within chain motifs has been analysed from three different points of view: three-body delocalization between C=C-C, two-body hydrogen bond delocalization indices and also between fragments (monomers). A many-body contribution to the interaction energy as well as electron density helps to establish the assistance of resonance in the strength of hydrogen bonds upon the formation of the present molecular chains. The direct relation between interaction energy and delocalization supports the original concept, and refutes some of the criticisms of the RAHB idea.

## 1. Introduction

The hydrogen bond is one of the most important noncovalent interactions that occur in nature. This is due to the fact that it plays a crucial role in various biological and physical processes, is important in material science, and is responsible for the stabilisation of macromolecular systems, including proteins, DNA, and molecular crystals [1,2,3,4,5,6,7,8]. Researchers are particularly interested in the strongest H-bonds, since they have the largest influence on the interacting molecular fragments. These strongest H-bonds are usually assisted by some additional effects. For instance, the so-called charge-assisted H-bonds (CAHBs) [9,10,11,12,13,14,15] may link molecular fragments, with their strength being sometimes close to that of covalent interactions, which, for instance, take place in the so-called salt bridges, CAHB(+/−) [16], mostly due to their dominant electrostatic contribution to the interaction energy [17]. Another type of H-bond assisted by additional effects is the polarisation-assisted H-bond (PAHB), in which the additional strengthening of the H-bridging occurs due to the additional polarisation of interacting fragments (molecules) and the presence of adjacent molecular entities. A good example might be an ice structure in which water molecules polarise each other, enhancing the H-bonding. Studies by Xantheas show that such enhancement (reflected by the non-additive contribution to interaction energy) is probably a half of the individual isolated H-bridge energy [18]. Last but not least, there is a class of H-bonds that is of our current interest in this paper: the so-called resonance-assisted H-bonds (RAHBs).

### 1.1. The Original Concept of RAHB

**Scheme 1:**
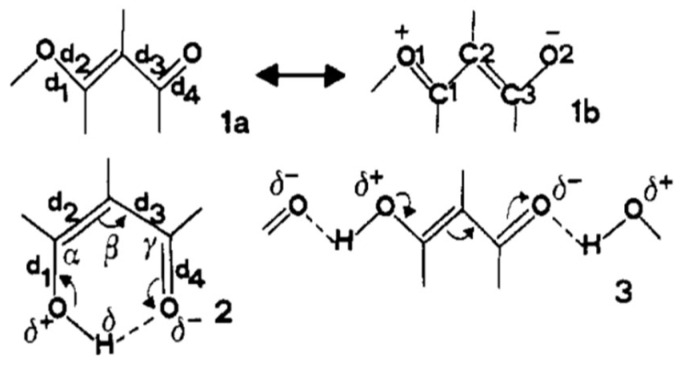
The scheme of β-diketone (1a) in enol form (1b). Two possible cases, intramolecular (2) and intermolecular H-bonds (3), are supposed to be resonance assisted. Reprinted with permission from Gilli, G.; Bellucci, F.; Ferretti, V.; Bertolasi, V. *J. Am. Chem. Soc.* **1989**, *111*, 1023–1028. Copyright 1989 American Chemical Society.

The paper that outlined the original concept of the resonance-assisted hydrogen bond was published by Gastone Gilli and his co-workers in the *Journal of the American Chemical Society* in 1989 [19]. The idea was to refer to structural changes in the conjugated β-diketone fragment in its enol form due to the formation of the O-H···O hydrogen bond. On the first page of the original paper by Gilli et al., the concept of RAHBs referred to both intramolecular and intermolecular H-bonds, as shown in the scheme taken from the original source (Scheme 1).

Generally speaking, according to the RAHB concept, the contribution of the resonance structure with the separated charges, 1b in Scheme 1, favours the formation of H-bonding. This is due to the presence of the formal positive charge on the donating oxygen atom in the hydroxyl group (which should increase the proton-donating properties of that centre because of the local electron deficit) and the formal negative charge on the accepting oxygen atom in the keto-fragment (which should, in turn, increase the proton-accepting properties of that centre due to the local surplus of electron charge). The contribution of structure 1b to the H-bond shall be manifested by the structural changes resulting from the formation of the H-bond. Therefore, “… *the idea of the work came from the empirical observation that a greater delocalization of the π-conjugated system occurs in* HOCR=CR-CR=O *fragment when it forms either intramolecular or infinite-chain intermolecular hydrogen bonding*” [19]. The simple analysis of CO and CC bonds in the conjugated fragment allowed Gilli et al. to point out that there is a direct relationship between the structural changes in that fragment and the H-bond strength. The formation of the H-bond results in the elongation of formally double CC and CO bonds and, at the same time, the shortening of their formally single counterparts. Reported empirical observations (crystallographic data, including the authors’ own results and CSD collection) were supported by semi-empirical calculations for the finite model of intramolecular RAHBs in a molecule with a malondialdehyde enol form (see Scheme 1).

**Scheme 2:**
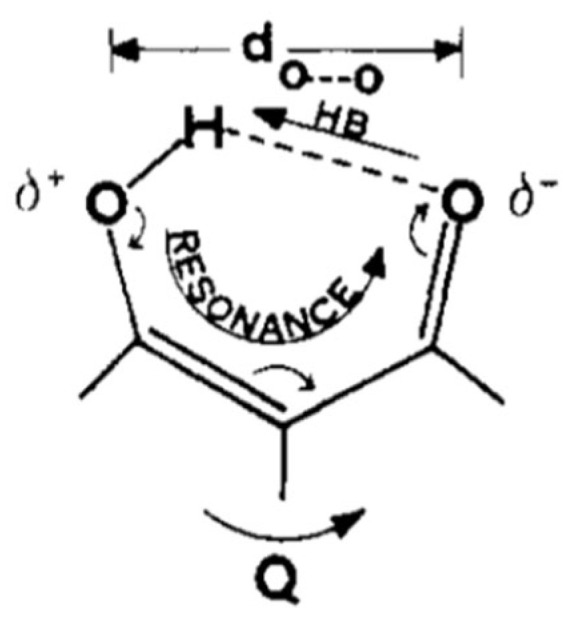
A graphical definition of RAHB. Reprinted with permission from Gilli, G.; Bellucci, F.; Ferretti, V.; Bertolasi, V. *J. Am. Chem. Soc.* **1989**, *111*, 1023–1028. Copyright 1989 American Chemical Society.

Later, Gilli and co-workers published an excellent collection of articles devoted to the topic of RAHBs [20,21,22,23,24,25,26,27,28,29,30,31,32,33,34,35]. What is important (not only in the context of our current contribution but also in the context of the criticism of the RAHB concept, which will be briefly introduced later), among this collection of 16 articles, with the exception of three citations [22,25,28], all the investigated systems correspond to intramolecular H-bonds, mostly those with the molecular pattern shown in Scheme 2 and its heterocyclic variations. The topic of RAHBs has been thoroughly reviewed, among other aspects of H-bonding theory, by Gilli and Gilli themselves [36].

### 1.2. The Criticism of the RAHB Concept

**Scheme 3:**
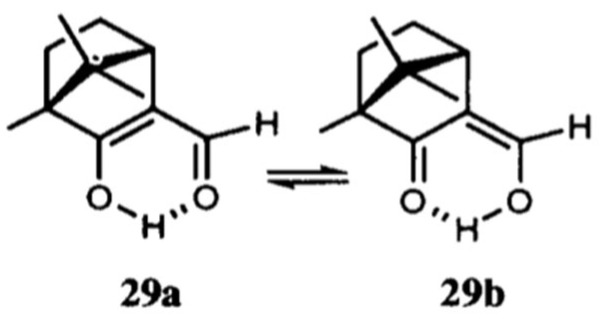
An example of a tautomer investigated in the context of the Mills–Nixon effect by Ramos et al. [37]. Original scheme from there.

Eight years after the first RAHB Gilli paper [19], Ramos et al. reported results of their analysis of β-dicarbonyl derivatives (analogues of the Scheme 2 system) [37]. Comparing the isomers, such as those shown in Scheme 3, they observed preferences in the H-bond formation which were correlated with the structure of the molecular skeleton bearing the H-bonded quasi-ring.

Ramos et al. explain H-bonding preferences in a system similar to that in Scheme 3, with a shorter H-bond in isomer (a), which is distinct to (b) due to a shorter CC bond placed in a quasi-ring opposite to the H-bond, referring to the Mills–Nixon effect [38]; that is, the structural influence of the rigid structure of some carbon molecular skeleton on the forced localisation of the delocalised, formally aromatic CC bonds. Therefore, no idea of the resonance effect is applied here. We consider this contribution by Ramos et al. to be the first trace of RAHB concept criticism. In fact, in 2004 a paper appeared in which the RAHB concept was questioned again, this time explicitly [39]. In this paper, a detailed analysis of the NMR properties obtained for systems that were an analogue to that in Scheme 2 allowed the authors to conclude that neither the coupling constants nor the proton chemical shifts provided any evidence for the existence of RAHBs. As the authors say “*…the enhanced stability of the IMHB in the unsaturated compound is associated with the sigma-skeleton of the molecule that allows the oxygen atoms to be in closer proximity than in the saturated analogue*”. Regarding RAHB concept counterpoints, it is worth mentioning an interesting contribution by Guevara-Vela et al. [40], who apply the quantum theory of atoms in molecules (QTAIM) [41] and derived from it the concept of interacting quantum atom (IQA) energy partitioning to explain the relation between H-bonding and π-electron delocalization along the quasi-ring in systems analogous to that in Scheme 2. The general conclusion seems to be against the Gilli RAHB concept. To quote the authors: “*As opposed to the usual description of RAHB interactions, these HBs lead to a larger electron localisation in the system, and concomitantly to larger QTAIM charges, which in turn lead to stronger electrostatic, polarization, and charge transfer components of the interaction*”. Since the paper by Ramos et al. [37], several articles have appeared in which the RAHB concept has been questioned more or less explicitly. See, for example, [39,42,43,44,45,46,47]. There were also many in which this idea was followed. See, for some recent examples, [48,49,50,51,52,53,54,55,56,57,58,59,60], and references therein.

However, it should be stressed that the criticism of the RAHB concept [39,42,43,44,45,46,47] was built mainly on the basis of intramolecular H-bonded models. It is also known that the IMHB interaction energy, the most basic physical parameter of any interatomic interaction, cannot be estimated explicitly. It can merely be evaluated approximately using various models, among which many were recently investigated in the context of methodological considerations by Jabłoński [61] and earlier by Sadlej et al. [62] (see also ref. [63]). To the best of our knowledge, there is only one paper in which the concept of RAHBs is addressed for an infinite H-bond pattern; that is, the paper by Trujillo et al. [64], where the authors, based on their previous contributions where the concept of RAHBs was questioned, extend their studies to open-chain structures. One of their main conclusions was that the intermolecular RAHBs exhibits a more significant effect of cooperativity with respect to their intramolecular counterparts due to conjugation.

Here, we revisit the original concept of RAHBs, examining it from the perspective of the many-body interactions approach and electron density delocalization analysis, referring directly to the interaction energy, as explicitly estimated, with π-electron conjugation measured explicitly using multicentre delocalization indices. For this reason, we use the open-chain motifs of RAHBs taken from a selection of crystal structures. We examine them against their non-conjugated counterparts in order to estimate the contribution from the π-electron conjugation.

## 2. Computational Methods

**Scheme 4:**
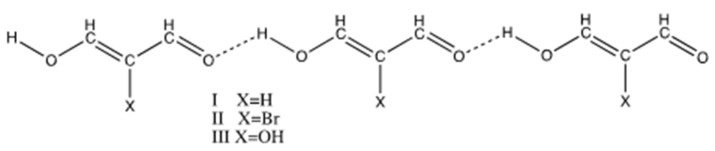
General structure of three-membered chain, where I: X=H, II: X=Br, III: X=OH.

All molecular systems taken from crystals (based on Scheme 4, named **I**, **II**, and **III**) were examined using the ωB97XD [65] variant of density functional theory with the aug-cc-pVTZ basis set [66,67,68]. This level of theory gives very good results in the case of hydrogen bonds or rather weak interactions with the contribution of dispersion effects, and it is appropriate for the calculation of relatively large systems [69,70]. All three systems (I-III) were calculated with single point calculations in geometries taken from the crystal state. The positions of hydrogen atoms involved in hydrogen bonding were normalized according to neutron diffraction data [71]. To account for geometry relaxation, system I was optimized using the same variant of the density functional theory ωB97XD, but with the 6-31++G(d,p) basis set [72]. To account for the π-electron delocalization, both unsaturated (**IV**) and saturated (**V**) chains were taken into account. Because in the crystals the chain of molecules lays in one plane, in these structures (**IV**,**V**), we also imposed such an arrangement (Cs) of the molecules in order to exclude cluster-like geometry. In the next step, both systems were recalculated with a single point calculation at the ωB97XD/aug-cc-pVTZ level of theory for further analysis. All the calculations were carried out using the Gaussian09 package (revision D.01) [73]. The atoms in molecules (AIM) methodology [41] was used to analyse the electron density of the systems using the AIMAll [74] and ESI-3D [75,76] programs. For all calculations, the Poincaré–Hopf relationship was satisfied.

In order to determine the interaction energies ($E_{int}^{SM}$) in the investigated complexes, we applied the supermolecular (SM) approach, in which the interaction energy of the complex is defined as the following sum:

(1) $$E_{int}^{SM}=E_{int}^{SM}\left[2-b\right]+E_{int}^{SM}\left[m-b\right]$$

where the first term denotes the two-body (pair) additive contribution and the second one indicates the many-body non-additive contribution to $E_{int}^{SM}$. Equation (1) is valid for a complex composed of six species, as in this case. For the SM approach, the above-mentioned contributions are expressed as:

(2) $$E_{int}^{SM}\left[2-b\right]=\overset{n-1}{\underset{i=1}{\sum }}\overset{n}{\underset{j=i+1}{\sum }}\left(E\left(i,j\right)-E\left(i\right)-E\left(j\right)\right)$$

(3) $$E_{int}^{SM}\left[m-b\right]=E_{int}^{SM}-E_{int}^{SM}\left[2-b\right]$$

where E(i,j) and E(i) represent the energies of a dimer and a monomer, respectively (i,j = 1, 2, 3, …, 7 and i < j to prevent repetition of terms, while 2-b and m-b refer to contributions of the two-body and many-body, respectively). Note that the dimers and monomers were in the same geometries as those in the six molecules complex (chain); thus, we exclude the deformation energy from our consideration. According to the many-body interaction approach, the interaction energy can be separated into its additive (two-body) and non-additive contributions (Equations (2) and (3)). Here, the latter is understood as a many-body contribution consisting of the sum of all contributions from three-body to n-body, depending on the number of bodies (molecules), *n*, in the molecular chain. For more about the many-body approach, see a collection of papers and references therein [77,78,79,80,81]. There are a few extensions of BSSE correction (basis set superposition error) to the many-body approach, but in general they give several numerical problems, including negative values of some n-body BSSE corrections. See paper [82] for an example analysis. For that reason, we do not apply a correction for BSSE in our energy analysis. It should be mentioned, however, that for the level of theory used by us, in particular the relatively large basis set, the expected BSSE will be in practice insignificant. To check it, we did calculations for the dimers of I, II and III, and corrections are 0.21, 0.24 and 0.30 kcal/mol, respectively. For that reason, we exclude BSSE correction from our analysis.

Delocalization indices (DI) are among the most popular bonding indicators. They provide quantitative information on the electron density shared between two atoms/fragments A and B [83]. The DI between atoms A and B, δ(A,B), is obtained by the double integration of the exchange-correlation density, $\Gamma_{\mathrm{xc}}\left(\vec{r_{1},}\vec{r_{2}}\right)$, with their respective atomic domains Ω_A_ and Ω_B_, and this is expressed as:

(4) $$\delta \left(A,B\right)=-2\int_{\Omega_{A}}\int_{\Omega_{B}}\Gamma_{XC}\left(\vec{r_{1}},\vec{r_{2}}\right)d\vec{r_{1}}d\vec{r_{2}}$$

In the particular case of a single-determinant closed-shell wavefunction, the DI can be expressed solely in terms of the elements of the atomic overlap matrices S(A) on the molecular orbital basis according to:

(5) $$\delta \left(A,B\right)=4\overset{occ}{\underset{ij}{\sum }}S_{ij}\left(A\right)S_{ij}\left(B\right)$$

where the summation runs over all doubly occupied molecular orbitals. In this work, the atomic partition is defined based on the condition of the zero-flux gradient in the one-electron density, following the quantum theory of atoms in molecules (QTAIM) [41]. Multicentre delocalization indices, δ(A_1_,…, A_n_), defined by Giambiasi [84], have been used to account for electron fluctuation between the atomic population of different three-centred fragments (C-C=C and H···O-H). δ(A_1_,…, A_n_) gives a measure of how the electron distribution is skewed from its mean. All the different electronic delocalization indices have been calculated using the ESI-3D program by Matito [75].

## 3. Results and Discussion

### 3.1. Intermolecular RAHB in View of Many-Body Interaction Theory

**Figure 1:**
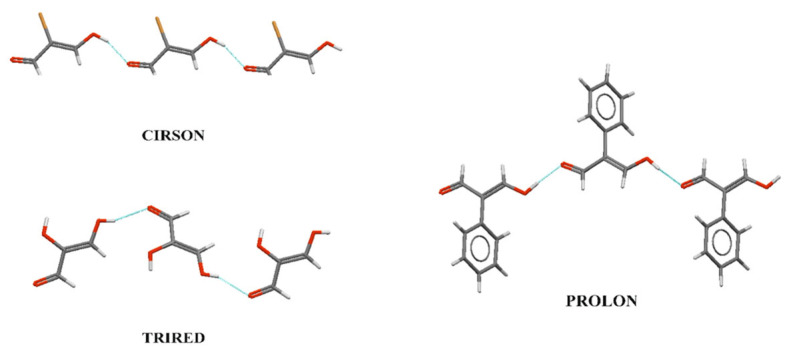
Fragments (three monomers) of RAHBs in crystal structures. Graphics taken from the CSD database [84] (CIRSON and TRIRED correspond to the investigated systems **II** and **III**, respectively).

In the first step, a search has been carried out through CSD [85] to find open patterns of RAHBs. The main criterion was the motif of the proton-donating and proton-accepting centre, conjugated via a sequence of formally double and single bonds, forming an intermolecular H-bonded pattern of a chain. A first obvious choice would be the moiety of malonaldehyde, eventually substituted on the carbon fragment, since pure malonaldehyde does not exist as a conjugated tautomer in the solid state (i.e., spontaneous tautomerisation onto malondialdehyde). After a detailed analysis of the CSD collection, we found three promising crystal structures, shown in Figure 1 (Refcodes: TRIRED, CIRSON and PROLON). In all cases, a pattern of intermolecular RAHBs forming an infinite chain was found. The geometrical parameters suggested the presence of relatively strong H-bonds (d_H···O_ distances in the range of 1.712–1.959 Å, while the corresponding sum of the van der Waals radii is 2.72 Å). To obtain an insight into energy characteristics of the RAHBs found in those crystal structures, we selected complexes consisting of *n* molecules that form RAHB-stabilized chains, where *n* was up to 6.

After the normalization of the covalent bonds according to their neutron diffraction lengths, the geometries obtained have been used for single point DFT calculations [71]. However, it was not possible to obtain the energy parameters at the applied level of calculation for the model system built on the crystal structure with recode PROLON; that is, the one with a large phenyl ring. That was due to SCF convergence failure for chains longer than three molecules. In case of structure **I**, the model has been simplified and the bromine atom has been substituted with a single H atom, keeping the rest of the molecule without geometrical changes. Since the structural situation found in the crystal does not necessarily have to correspond to that which would exist in the isolated model, additionally representative models have been used for calculations that include geometrical optimisation.

As one of the main objectives of the present work is to study the importance of π-electrons in the RAHBs for linear intermolecular chains, we design two more models. The first was the malonaldehyde model that forms the linear complex stabilized by the intermolecular RAHBs due to the possibility of π-conjugation along the sequence of formally single and double bonds within each individual molecule within a chain, referred to as system **IV**. This is in fact system **II**, but with its optimized geometry and the simplified counterpart of the interaction pattern found in crystal structures **II**. The second model chosen for the optimization procedure, **V**, was the saturated counterpart of **IV**, and thus the one in which the pattern of interaction has been kept, but in which no π-conjugation can occur. This is the obvious reference case for when the H-bond is not assisted by the resonance effect. From now on, five different structures will be studied, **I**, **II** and **III**, with frozen geometry based on crystal structures, while **IV** and **V** are fully optimized H-bond-stabilized chains (with up to six molecules).

Table 1 shows the energetic parameters associated with the different systems. The interaction energy, two-body interaction energies, and many-body interaction energies per molecule are listed for each chain and for each size. In general, when comparing the data in Table 1, one may immediately notice very similar results in the case of all conjugated systems (**I**–**IV**). It is worth mentioning that, according to QTAIM topology, in III, there is an extra H-bond of the C-H…O type, which shall have its own contribution to the interaction energy. However, comparing interaction energy parameters (Table 1) and QTAIM parameters at BCPs (Table S1 and Figure S17), this contribution is rather small. We can see that the total interaction energy obviously increases with the increasing number of molecules in the chain, and, at the same time, it increases per molecule. For two molecules, the interaction energy is, in all cases, about 13 kcal/mol. One of the most informative parameters will be the interaction energy (total, two-body and many-body) when the chain is increasing to infinite. In all cases, the relation between the total interaction energy $E_{int}^{SM}$ per chain and the number of molecules present in the chain, n, will excellently fit to linear regression:

(6) $$E_{int}^{SM}=an+b$$

where n is the number of molecules in the chain.

This linear equation can be modified to estimate the extrapolation of the interaction energy per molecule for an infinite number of molecules. When estimating the interaction energy per molecule, one should divide both sides of Equation (6) by n, obtaining a new energy function, interaction energy per molecule $E_{int/mol}$, defined as:

(7) $$E_{int/mol}^{SM}=a+\frac{b}{n}$$

and since $\underset{n\to \infty }{lim}E_{int/mol}^{SM}=a$, in Equation (1), the slope of the line is in fact the extrapolation of $E_{int/mol}^{SM}$, which is explicitly estimated for an infinite number of molecules in the chain (n→∞). This energy measure is shown in the last column of Table 1. The rest of parameters related to the different linear regression are collected in Table S2 in the Supplementary Materials, together with graphical representations of the interaction energy and its components, estimated as a function of the number of molecules in the chain (Figures S2–S16).

As can be seen, in conjugated systems, the total interaction energy per molecule oscillates around 17 kcal/mol. The largest value was found for **IV**, since this is an optimized geometry system, and thus it has the local minimum energy. The two-body contribution to the total interaction energy, in general, follows its parent parameter and oscillates around 14 kcal/mol. Clearly, both the total and two-body interaction energy estimated for the non-conjugated system **V** is significantly smaller, being almost half of that found for **I**–**IV**. Therefore, a partial conclusion can be formulated, according to which the explicitly estimated interaction energy is undoubtedly larger in RAHB patterns when compared with its non-resonance assisted counterparts. This is true for intermolecular interactions, so an intramolecular effect (steric effect, H···O bond forced to be shorter, etc.) cannot be considered here as a reason for the stronger H-bonding of the conjugated systems.

Interestingly, as they are evidently distinct to non-conjugated system **V**, in the case of all conjugated systems, there is a significant contribution from the many-body component of the interaction energy. This parameter appears as an effect of the influence of the neighbour environment on a given energy parameter (the interaction here) and is a non-additive contribution to the total interaction energy. Here, it should be interpreted as the evidence of a synergism of the individual H-bridges present in the given chain. The same parameter in **V** is very close to zero; thus, in this case, such synergism is insignificant. One may expect that the presence of adjacent molecules (which are dipoles individually) additionally favours contributions from resonance charge-separated structures in the given considered molecule. Obviously, such an effect cannot be present in a non-conjugated molecular system. Therefore, the next partial conclusion can be as follows: the π-conjugation in **I**–**IV** allows the additional enhancing of the H-bonding due to the many-body effects. This effect does not occur in non-conjugated systems. Is this in fact due to the resonance effect? In order to answer this question, we performed an analysis of the π-electron effects in the investigated systems, using π-electron delocalization indices.

### 3.2. The Resonance in Intermolecular RAHB

One of the partial conclusions that arose in the previous analysis was related to π-conjugation in **I–IV** systems, which allows the enhancement of H-bonding. These effects, which were not found in system **V** (with no π-electrons), suggests that resonance effects are important in these chains. Therefore, to answer this question, we analysed the delocalization of the π-electrons within the different systems, together with the charge polarization due to the synergies caused by the different monomers.

In the next section, we will analyse the electron density delocalization based on three different points of view. First, we will focus on the three-body delocalization between C=C-C, as well as the hydrogen bond delocalization indices. A second step will be the analysis of the delocalization between neighbouring fragments while the chain is increased, ending with the change of the atomic charges in the frontier hydrogen bond atoms. These different parameters will help us to understand the role of π-electrons in enhancing the strength of HB in saturated and non-saturated systems. As the delocalization indices are very dependent on the bond length, the main conclusions will be based on systems **I**, **II**, and **III**, which present the common bond length. For these systems, the many-body resonance effect will be based again on three-body electron delocalization, followed by different bond delocalization indices and a final fragment delocalization analysis. At the same time, the natural population of the atoms involved in the HB will be taken into account.

To analyse how the delocalization within the C=C-C fragment changes, the three-body MCI has been calculated for each fragment in the different chains (from one to six monomers), with results shown in Table 2. As was explained, **IV** and **V** were fully optimized, so the C-C bonds are slightly different for the different monomers in the same chain. Although these multicentre indices depend on the distances, the change is so small that they can still be used to show a first picture of the delocalization along the CCC fragment following the increase in the number of the chains. Let us start with system **IV**, with only one monomer, and with an MCI(C=C-C) of 0.1036 au. The addition of a second monomer leads to an increase in the degree of delocalization within the C=C-C fragment in both monomers, which is larger in the first one (0.1168 au and 0.1076 au; note that we keep the same convention through the whole manuscript, according to which the first is the one on the left end of the chain). This trend is shown along the whole series: the addition of a new monomer increases the delocalization along C=C-C (at a maximum of 0.1269 au) in the rest of the monomers, although the first one is up to five monomers away. When one analyses the same MCI(CCC) for the saturated chains (system **V** in Table 2), it can be observed that there is almost no change when adding new monomers (changing from 0.0302 to 0.0310). From these first data, we can corroborate that there is an increase in the amount of π-conjugation in the C=C-C fragment, which, of course, is not found in non-conjugated systems.

Systems **I**, **II** and **III** are calculated with frozen geometry (see Scheme 4), so there is no bond length dependence on the different indices. All three chains present similar behaviour compared to the previous conjugated system (**IV**). Although the change is smaller, as we enlarge the system, the delocalization within the C=C-C increases, going from 0.1030 au for the monomer in system **II** up to 0.1194 au for the central monomer in the six-membered chain. All of them follow the same pattern, with the delocalization for system **II** (X=H) larger than those for systems **I** and **III**. For the six-membered chain, the monomers in the centre are always the ones with the larger delocalization.

Now, we need to find out whether there is a relation between this enlargement of the delocalization within each monomer and the enhancement of the hydrogen bond strength. Following the previous analysis, one could think that the three-body delocalization between H··OH should give us the answer. In Table S8, the three-body MCI(H···OH) are collected for the different chains. There is no increase in the degree of delocalization when more monomers are added, but it is worth mentioning that the indices are so small that they can hardly be significant. On the other hand, the fact that it is not increasing is related, meaning that the strength of this HB is surely not related to a three-body problem (H···OH), but to a two-body problem; that is, delocalization within the hydrogen bond (O···H).

In Table 3, the delocalization indices for O···H and HO (δ(O···H), δ(HO)) are shown, while the indices for the rest of the bonds, that is, C=C and C-C, can be found in the Supplementary Materials, Tables S14–S18. It is known that O···H strength can be related to DI [81], so their values are very dependent on the HB distances. Therefore, we will focus mainly on systems **I**, **II**, and **III**, as they have the same frozen HB length. When no substituent is added (system II), the δ(O··H) for the dimer is 0.0990 au, increasing to 0.0999 when a second monomer is added, and going up to 0.1019 for the six-membered chain (with its central hydrogen bond). This increase in the δ(O···H) (e.g., 3%) is directly related to an increase in the HB strength, and it is found in all the hydrogen bonds along the chain. Again, if we now check the electron delocalization between the HBs within the longer chains (n = 6), we can see that the central hydrogen bond is the one with the largest delocalization, representing the strongest one. However, the HO bond DI (δ(HO)) always decreases while the size of the chain increases. If we now focus on the C=C and C-C bonds (Tables S9–S13), we can assess the increase in the degree of delocalization within the C=C-C fragment associated with the number of monomers. This can be translated into a decrease in the double bond character of C=C (for system I, Table S8, it goes from 1.5255 au to 1.4593 au), and an increase in the double bond electronic character of C-C (from 1.0548 au for n = 1 to 1.1145 au for n = 6). For the planar systems (that is, **I** and **II**), the delocalization indices have been separated into contributions σ and π. Therefore, for these systems, it can be observed that the main change is due to π-electron delocalization.

More information on the electron delocalization between the monomers can be found if we consider the entire electron delocalization between the fragments (not only between O···H). Table 4 shows the delocalization between the monomers for the different systems **I** to **V** when adding the monomers up to six. An increase in the amount of electron delocalization between the monomers is observed as we enlarge the chain, again, with a greater amount of delocalization within the central monomers. Systems **III** presents higher values as a result of the extra interaction found between the monomers because of the OH substituent. It is worth mentioning that for system **IV** (with its whole optimized geometry), the change in the amount of delocalization is also higher, following the expected trend, while for system **V** (no π electrons available), the values are much smaller, and they do not follow any trend.

**Figure 2:**
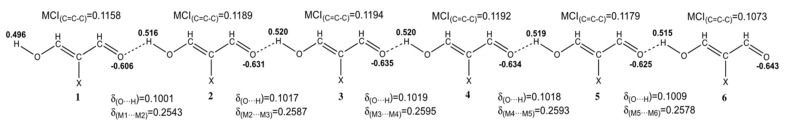
NPA charges, δ(O··H) and δ(M1-M2) and three-body MCI(C=C-C) for system II.

As well as the increase in the amount of electron delocalization between monomers when the chain is increased, a change in the polarity of the atoms forming the HB will also need to be taken into account. In the Supplementary Materials, all data are shown for the five different systems (Table S19), while in Figure 2, the atomic charges for system **II** are depicted. From the data, it can be observed that there is an increase in the degree of polarity of O···H while the chain increases. Moreover, for the six-membered chain, the central monomer presents the hydrogen with a higher proton character. When the saturated and unsaturated systems (**V** and **IV**, respectively) are compared, one can observe that the change in charges is not so different, with each giving the same amount of synergy between the monomers. From these data, it can be assessed that the increase in the degree of polarity between the monomers as the chain increases is not responsible for the difference between the saturated and unsaturated chains. The difference between both systems arises from electron delocalization within the chain.

All the previous data can be summarized in Figure 2, where the data for system number **II** are depicted. This shows how the C=C-C three body MCI increases in the central part of the chain, as well as in the delocalization indices. The NPA charges for the atoms involved in hydrogen bond formation are also represented, with the more polarized bonds being the most central ones.

From the point of view of π-electron delocalization, an increase in the degree of electron delocalization within the C=C-C bond is found as we add more monomers. At the same time, the hydrogen bonds (O···H) also show a more delocalized electron density, because they are always higher in the central part of the molecules. These effects, together with the increase in the amount of delocalization between the fragments (monomers), help to show that the resonance effect helps to increase the hydrogen bond strength, thus confirming that they are assisted by resonance (RAHB).

## 4. Conclusions

We have investigated the strength of hydrogen bonding for selected systems taken from crystal structures, which are the open-chain motifs of proton donating and accepting centres coupled through a sequence of formally double and single bonds, forming the H-bonds. Additionally, fully optimized systems corresponding to those found in the experiment were also investigated. We noticed that the total interaction energy obviously increases with the increasing number of molecules in the chain (from one to six monomers), and at the same time the interaction energy increases per molecule. In conjugated systems, the total interaction energy per molecule oscillates around 17 kcal/mol, while the two-body contribution in general follows its parent parameter and oscillates around 14 kcal/mol. Clearly, both the total and two-body interaction energy estimated for the non-conjugated system (non-RAHB) is significantly smaller at almost half that found for the conjugated systems. Therefore, the explicitly estimated interaction energy is undoubtedly larger in RAHB patterns when compared to its non-resonance-assisted counterparts, which present almost no contribution of many-body components. This parameter appears to be an effect of the influence of the neighbour environment on a given energy parameter (i.e., of the interaction here) and is a non-additive contribution to total interaction energy. Therefore, the π-conjugation allows the additional enhancement of the H-bonding due to many-body effects, which clearly do not occur in non-conjugated systems. From a straight mathematical relation between the interaction energy and its components and the number of molecules within a chain, the explicit estimation of energy parameters for the infinite chain has been given.

The analysis of electron density delocalization helped us to understand the role of π-electrons in enhancing the hydrogen bond strength in the investigated systems. The estimated three-body multicentre indices (MCI) of delocalization for each fragment in the different chains (from one to six monomers), corroborate that there is an increase in the amount of π-conjugation in the C=C-C fragment, which of course cannot occur in non-conjugated systems. The monomers in the centre are always those with larger delocalization. The two-body delocalization within the hydrogen bond (O···H) also increases with the increasing number of fragments within the chain, with the central hydrogen bond being the one with the largest delocalization, and, thus, the strongest one. When we consider all electron delocalization between fragments (not only between O···H) we see that an increase in the degree of electron delocalization between monomers is observed for the longer chains, again, with a larger amount of delocalization within the central part of the chain. No significant differences between the polarity of the atoms (atomic charges) forming the H-bond in saturated and unsaturated chains were found. Therefore, polarity is not the property directly responsible for the extra stabilization of RAHB.

## Figures and Tables

**Table 1:** Interaction energy, two-body interaction energy and many-body interaction energy per molecule (kcal/mol) for chains from n = 2 to n→∞.

System		2	3	4	5	6	n→∞
	$E_{int}^{SM}\;$	−12.89	−14.29	−15.01	−15.45	−15.73	−16.48
**I**	$E_{int}^{SM}\left[2-b\right]\;$		−13.24	−13.41	−13.52	−13.59	−13.77
	$E_{int}^{SM}\left[m-b\right]$		−1.06	−1.60	−1.93	−2.14	−2.87
	$E_{int}^{SM}\;$	−13.38	−14.83	−15.59	−16.05	−16.36	−17.14
**II**	$E_{int}^{SM}\left[2-b\right]\;$		−13.73	−13.90	−14.01	−14.08	−14.26
	$E_{int}^{SM}\left[m-b\right]$		−1.10	−0.68	−2.04	−2.28	−3.08
	$E_{int}^{SM}$	−12.92	−14.33	−15.12	−15.64	−15.95	−16.94
**III**	$E_{int}^{SM}\left[2-b\right]\;$		−13.26	−13.47	−13.61	−13.71	−14.04
	$E_{int}^{SM}\left[m-b\right]$		−1.07	−1.65	−2.03	−2.24	−3.47
	$E_{int}^{SM}$	−12.96	−14.99	−15.48	−16.21	−16.65	−17.89
**IV**	$E_{int}^{SM}\left[2-b\right]\;$		−13.51	−13.90	−11.69	−14.38	−14.84
	$E_{int}^{SM}\left[m-b\right]$		−0.98	−1.58	−2.03	−2.28	−3.27
	$E_{int}^{SM}$	−6.88	−7.71	−7.18	−7.15	−7.19	−7.30
**V**	$E_{int}^{SM}\left[2-b\right]\;$		−7.63	−6.94	−6.88	−6.89	−6.93
	$E_{int}^{SM}\left[m-b\right]$		−0.08	−0.24	−0.27	−0.30	−0.38

**Table 2:** Three-body MCI(C=C-C) (au) for **I**, **II**, **III**, **IV** and **V** chains, with #n being the number of monomers.

	#n	1	2	3	4	5	6
**I**	1	0.0925					
	2	0.1033	0.0952				
	3	0.1046	0.1060	0.0957			
	4	0.1050	0.1072	0.1065	0.0959		
	5	0.1051	0.1075	0.1077	0.1067	0.0960	
	6	0.1052	0.1077	0.1081	0.1079	0.1069	0.0960
**II**	1	0.1030					
	2	0.1137	0.1063				
	3	0.1152	0.1169	0.1070			
	4	0.1156	0.1184	0.1176	0.1072		
	5	0.1158	0.1188	0.1190	0.1178	0.1073	
	6	0.1158	0.1189	0.1194	0.1192	0.1179	0.1073
**III**	1	0.0947					
	2	0.1025	0.0999				
	3	0.1032	0.1077	0.1008			
	4	0.1035	0.1082	0.1085	0.1012		
	5	0.1036	0.1085	0.1090	0.1088	0.1013	
	6	0.1036	0.1086	0.1093	0.1093	0.1089	0.1013
**IV**	1	0.1036					
	2	0.1168	0.1076				
	3	0.1189	0.1222	0.1088			
	4	0.1198	0.1247	0.1236	0.1091		
	5	0.1202	0.1254	0.1264	0.1246	0.1093	
	6	0.1208	0.1256	0.1269	0.1269	0.1243	0.1056
**V**	1	0.0302					
	2	0.0308	0.0301				
	3	0.0308	0.0309	0.0302			
	4	0.0307	0.0308	0.0310	0.0341		
	5	0.0307	0.0308	0.0310	0.0342	0.0301	
	6	0.0307	0.0308	0.0308	0.0341	0.0308	0.0301

**Table 3:** DI for HB formation: δ(O··H) and δ(HO) (au) for systems **I**, **II**, **III**, **IV** and **V**, with #n being the number of monomers.

	#n	HO	O···HO		O···HO		O···HO		O···HO		O···HO	
**I**	1	0.6245										
	2	0.6132	0.0939	0.4523								
	3	0.6104	0.0945	0.4416	0.0949	0.4444						
	4	0.6094	0.0947	0.4389	0.0957	0.4336	0.0952	0.4423				
	5	0.6090	0.0948	0.4380	0.0959	0.4310	0.0959	0.4315	0.0953	0.4416		
	6	0.6088	0.0948	0.4376	0.0959	0.4300	0.0961	0.4289	0.0960	0.4307	0.0954	0.4412
**II**	1	0.6310										
	2	0.6197	0.0990	0.4603								
	3	0.6170	0.0999	0.4497	0.1004	0.4523						
	4	0.6161	0.1001	0.4473	0.1014	0.4417	0.1008	0.4504				
	5	0.6158	0.1001	0.4463	0.1016	0.4392	0.1017	0.4396	0.1009	0.4495		
	6	0.6156	0.1001	0.4460	0.1017	0.4384	0.1019	0.4372	0.1018	0.4389	0.1009	0.4492
**III**	1	0.6349										
	2	0.6223	0.0925	0.4653								
	3	0.6189	0.0937	0.4536	0.0934	0.4582						
	4	0.6176	0.0941	0.4506	0.0948	0.4463	0.0940	0.4558				
	5	0.6170	0.0941	0.4493	0.0952	0.4434	0.0952	0.4440	0.0941	0.4549		
	6	0.6167	0.0943	0.4489	0.0953	0.4421	0.0957	0.4410	0.0954	0.4431	0.0942	0.4545
**IV**	1	0.6161										
	2	0.6048	0.0957	0.4659								
	3	0.6011	0.0968	0.4548	0.1005	0.4526						
	4	0.6002	0.0993	0.4469	0.1017	0.4398	0.1064	0.4426				
	5	0.5990	0.1058	0.4373	0.1042	0.4343	0.1052	0.4333	0.1079	0.4409		
	6	0.5986	0.1091	0.4320	0.1145	0.4208	0.1039	0.4320	0.1043	0.4336	0.1063	0.442
**V**	1	0.6577										
	2	0.6535	0.0701	0.55								
	3	0.6522	0.072	0.5425	0.0722	0.5444						
	4	0.6517	0.0741	0.5391	0.0594	0.5556	0.0739	0.5397				
	5	0.6516	0.0735	0.5394	0.0626	0.5502	0.062	0.5499	0.0741	0.5395		
	6	0.6521	0.0732	0.5395	0.0701	0.5404	0.0579	0.5581	0.0624	0.5499	0.0728	0.5423

**Table 4:** Fragment MCI for **I**, **II**, **III**, **IV** and **V** chains, with #n being the number of monomers.

	#n	1–2	2–3	3–4	4–5	5–6
**I**	1	0.2845				
	2	0.2854	0.2884			
	3	0.2858	0.2897	0.2894		
	4	0.2860	0.2902	0.2906	0.2898	
	5	0.2861	0.2903	0.2912	0.2910	0.2899
**II**	1	0.2524				
	2	0.2537	0.2564			
	3	0.2541	0.258	0.2574		
	4	0.2543	0.2585	0.259	0.2515	
	5	0.2543	0.2587	0.2595	0.2593	0.2578
**III**	1	0.3056				
	2	0.3064	0.3092			
	3	0.3071	0.3106	0.3105		
	4	0.3072	0.3115	0.3110	0.3110	
	5	0.3075	0.3116	0.3129	0.3124	0.3112
**IV**	1	0.2279				
	2	0.2418	0.2449			
	3	0.2461	0.2623	0.2573		
	4	0.2559	0.2697	0.2717	0.2612	
	5	0.2609	0.2823	0.2752	0.2712	0.2585
**V**	1	0.1658				
	2	0.1692	0.1721			
	3	0.1745	0.1589	0.1717		
	4	0.1727	0.1635	0.1609	0.1719	
	5	0.1702	0.1705	0.1647	0.1658	0.1735

## Data Availability

Not applicable.

## Supplementary Materials

The following are available online at https://www.mdpi.com/article/10.3390/ijms23010233/s1.
