# Supplementary material for: Resonance-Assisted Hydrogen Bond—Revisiting the Original Concept in the Context of Its Criticism in the Literature
Source: Int J Mol Sci. 2021 Dec 26;23(1):233. doi: 10.3390/ijms23010233 (PMC8745518; doi:10.3390/ijms23010233)
Supplement: Supplementary file 1 [file ijms-23-00233-s001.zip › ijms-1502089-supplementary.pdf]

Resonance Assisted Hydrogen Bond - revisiting the original concept in the context of its criticism in the literature.

Małgorzata Domagała [1,]*, Sílvia Simon [2,]*, Marcin Palusiak [1]

[a] *Department of Physical Chemistry, Faculty of Chemistry, University of Łódź*
Pomorska 163/165, 90236 Łódź, Poland
[b] *Institut de Química Computacional I Catàlisi, Departament de Química, Universitat de Girona*
C/Ma Aurèlia Capmany, 69; 17003-Girona, Catalonia

Contents:

page S2 **Figure S1.** Graphical representation of investigated systems for 6 molecules for structures I-V.
page S2 **Figure S2.** Linear relation between E_{int} [kcal/mol] and number of molecules in structure I.
page S3 **Figure S3.** Linear relation between E_{int} [kcal/mol] and number of molecules in structure II.
page S3 **Figure S4.** Linear relation between E_{int} [kcal/mol] and number of molecules in structure III.
page S3 **Figure S5.** Linear relation between E_{int} [kcal/mol] and number of molecules in structure IV.
page S4 **Figure S6.** Linear relation between E_{int} [kcal/mol] and number of molecules in structure V.
page S4 **Figure S7.** Linear relation between E_{2-b} [kcal/mol] and number of molecules in structure I.
page S4 **Figure S8.** Linear relation between E_{2-b} [kcal/mol] and number of molecules in structure II.
page S5 **Figure S9.** Linear relation between E_{2-b} [kcal/mol] and number of molecules in structure III.
page S5 **Figure S10.** Linear relation between E_{2-b} [kcal/mol] and number of molecules in structure IV.
page S5 **Figure S11.** Linear relation between E_{2-b} [kcal/mol] and number of molecules in structure V.
page S6 **Figure S12.** Linear relation between E_{m-b} [kcal/mol] and number of molecules in structure I.
page S6 **Figure S13.** Linear relation between E_{m-b} [kcal/mol] and number of molecules in structure II.
page S6 **Figure S14.** Linear relation between E_{m-b} [kcal/mol] and number of molecules in structure III.
page S7 **Figure S15.** Linear relation between E_{m-b} [kcal/mol] and number of molecules in structure IV.
page S7 **Figure S16.** Linear relation between E_{m-b} [kcal/mol] and number of molecules in structure V.
page S7 **Figure S17.** Molecular graph of six-molecules chain of structure III.
page S8 **Table S1.** Selected QTAIM parameters [au] of six-molecules chain of structure III.
page S8 **Table S2.** Linear equation constants together with coefficient of determination to linear function, R^2, for total interaction energy and its two components, additive two-body and non-additive many-body contribution.
page S9 **Table S3.** Matrix of two-body contributions to E_{int} [kcal/mol] for structure I.
page S9 **Table S4.** Matrix of two-body contributions to E_{int} [kcal/mol] for structure II.
page S9 **Table S5.** Matrix of two-body contributions to E_{int} [kcal/mol] for structure III.
page S9 **Table S6.** Matrix of two-body contributions to E_{int} [kcal/mol] for structure IV.
page S9 **Table S7.** Matrix of two-body contributions to E_{int} [kcal/mol] for structure V.
page S10 **Table S8.** Three-body MCI(H···OH) (au) for I, II, III, IV and V chains, being **#n** the number of monomers.
page S11 **Table S9.** Total, σ and π contributions δ(C=C) and δ(C-C) for systems I.
page S12 **Table S10.** Total, σ and π contributions δ(C=C) and δ(C-C) for systems II.
page S13 **Table S11.** Total δ(C=C) and δ(C-C) for systems III.
page S14 **Table S12.** Total, σ and π contributions δ(C=C) and δ(C-C) for systems IV.
page S15 **Table S13.** Total, σ and π contributions δ(C=C) and δ(C-C) for systems V.
page S16 **Table S14.** Delocalization indices for HB formation: Total, σ and π contributions δ(O··H) and δ(HO) for systems.
page S17 **Table S15.** Delocalization indices for HB formation: δ(O··H) and δ(HO) for systems II being #n the number of monomers.
page S18 **Table S16.** Delocalization indices for HB formation: δ(O··H) and δ(HO) for systems III being #n the number of monomers.
page S19 **Table S17.** Delocalization indices for HB formation: δ(O··H) and δ(HO) for systems IV being #n the number of monomers. Total, σ and π contributions.
page S20 **Table S18.** Delocalization indices for HB formation: δ(O··H) and δ(HO) for systems V being #n the number of monomers. Total, σ and π contributions.
page S21 **Table S19.** NPA charges for the atoms involved in the RAHB for each monomer.
page S22 **Table S20.** Atom coordinates of examined complexes corresponding to single point geometries at ωB97XD/aug-cc-pVTZ level of theory (for structures I-III) for a chain of 6 molecules.
page S23 **Table S21.** Atom coordinates of examined complexes corresponding to optimized geometries at ωB97XD/6-31++G(d,p) level of theory (for structures IV-V).

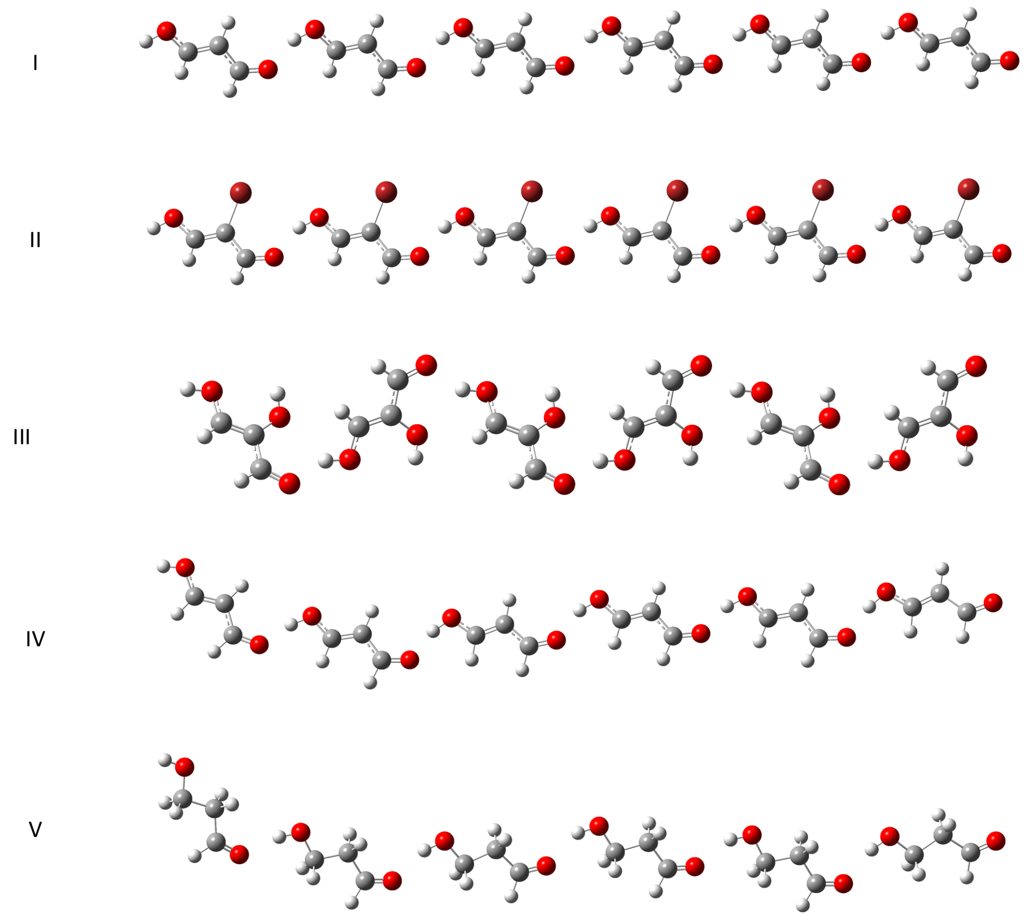

Figure S1. Graphical representation of investigated systems for 6 molecules for structures I-V.

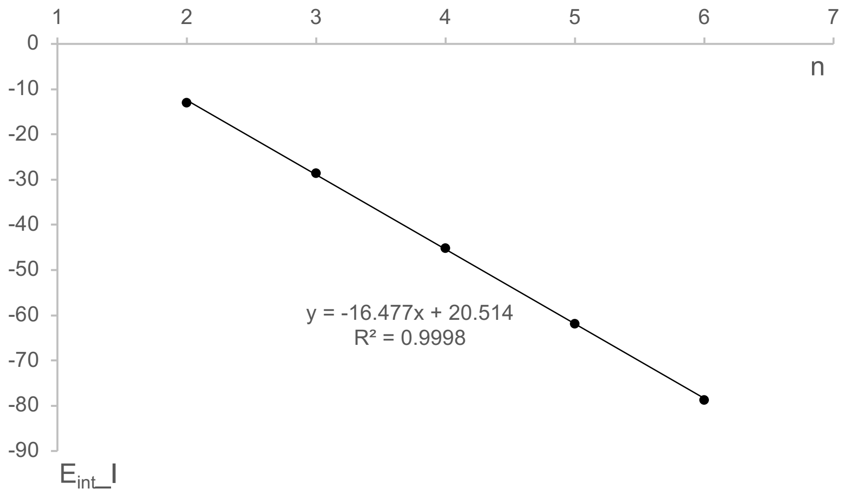

Figure S2. Linear relation between E_{int}^{SM} [kcal/mol] and number of molecules in structure I.

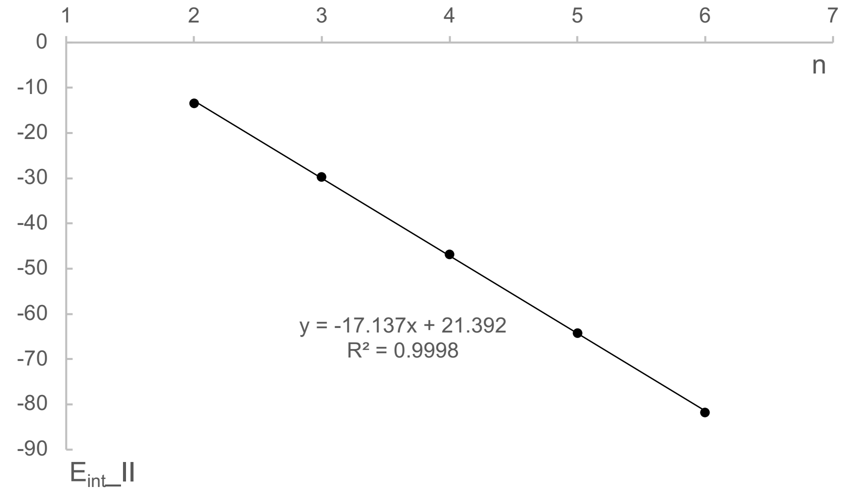

Figure S3. Linear relation between E_{int}^{SM} [kcal/mol] and number of molecules in structure II.

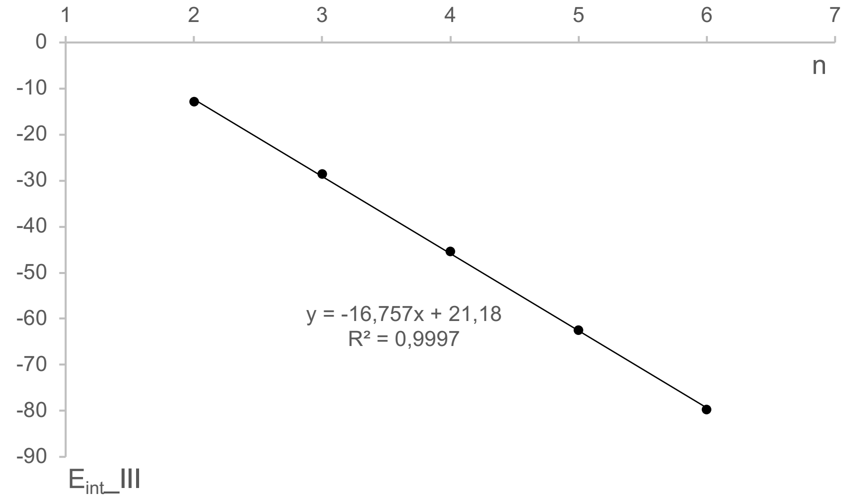

Figure S4. Linear relation between E_{int}^{SM} [kcal/mol] and number of molecules in structure III.

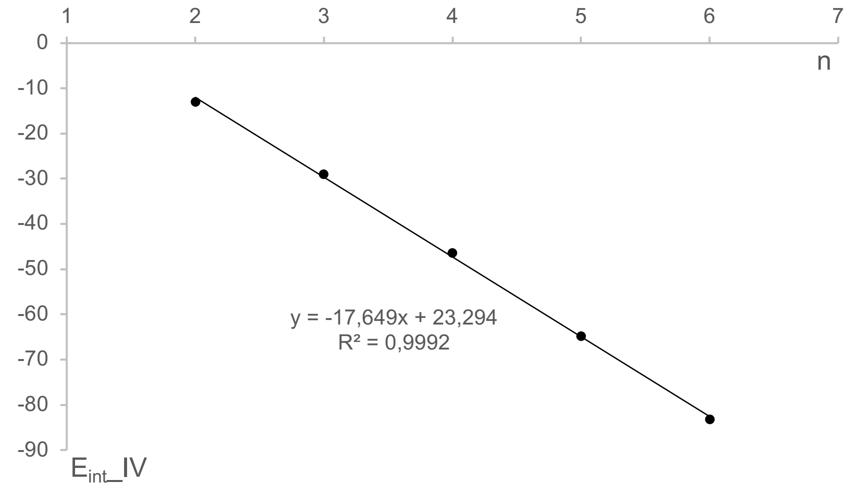

Figure S5. Linear relation between E_{int}^{SM} [kcal/mol] and number of molecules in structure IV.

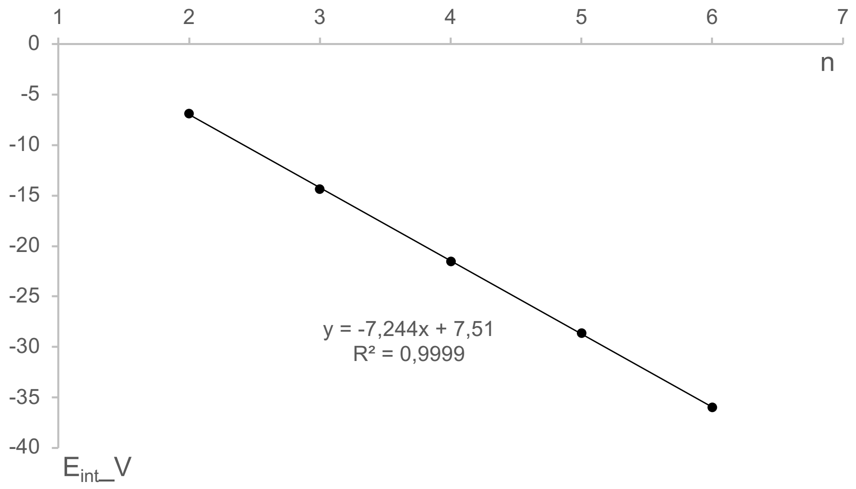

Figure S6. Linear relation between E_{int}^{SM} [kcal/mol] and number of molecules in structure V.

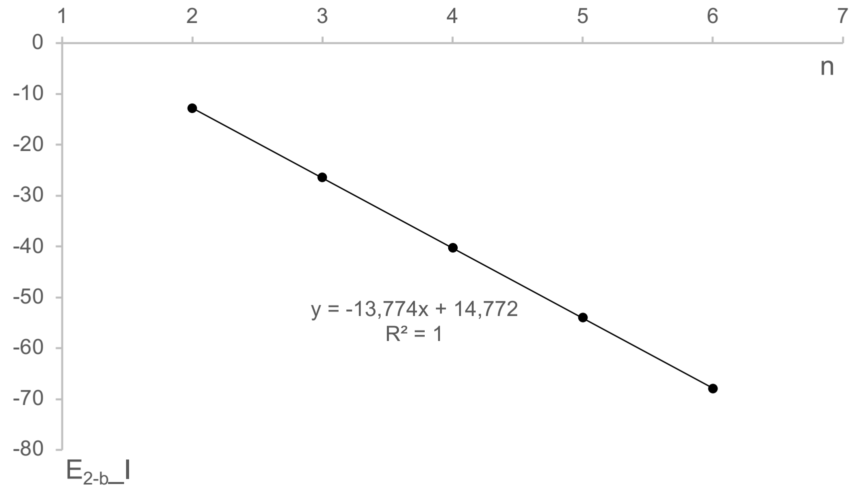

Figure S7. Linear relation between $E_{int}^{SM}[2-b]$ [kcal/mol] and number of molecules in structure I.

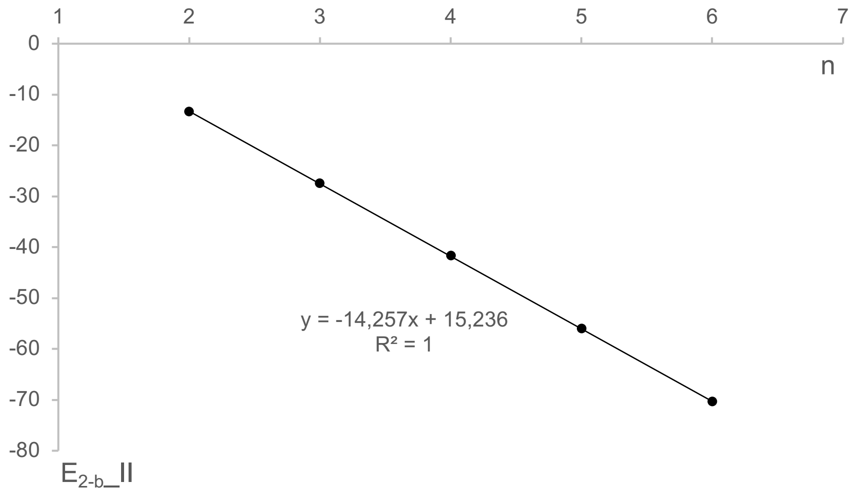

Figure S8. Linear relation between $E_{int}^{SM}[2-b]$ [kcal/mol] and number of molecules in structure II.

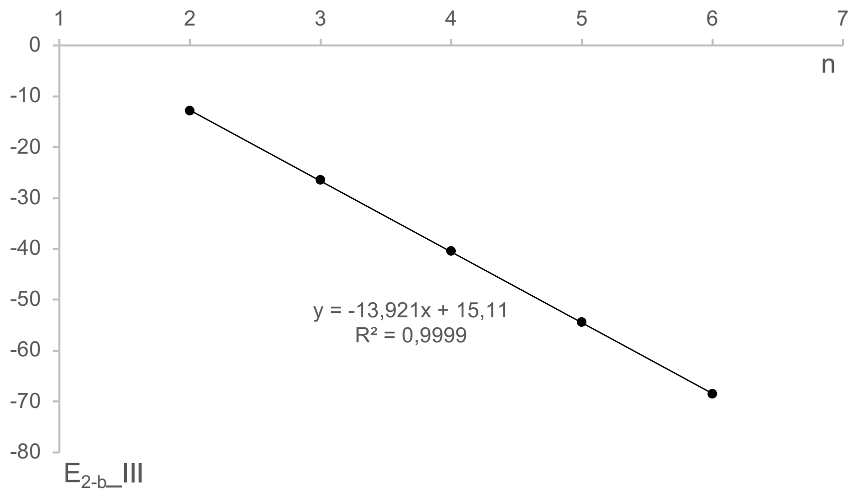

Figure S9. Linear relation between $E_{int}^{SM}[2-b]$ [kcal/mol] and number of molecules in structure III.

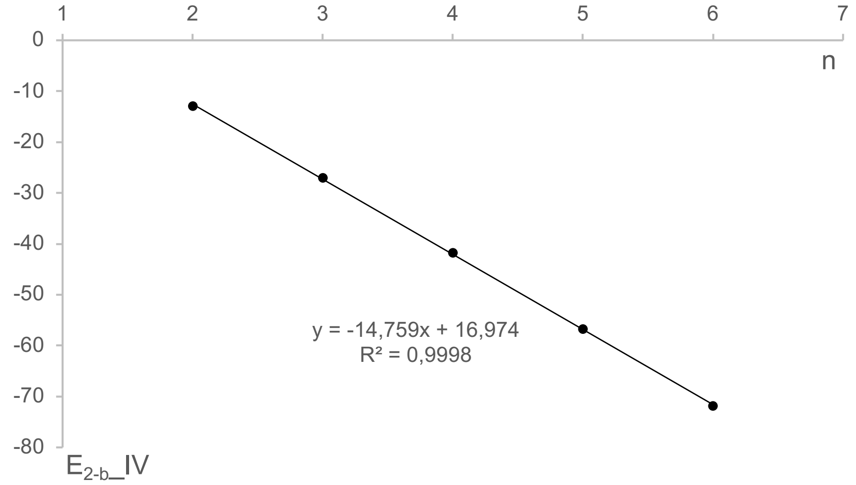

Figure S10. Linear relation between $E_{int}^{SM}[2-b]$ [kcal/mol] and number of molecules in structure IV.

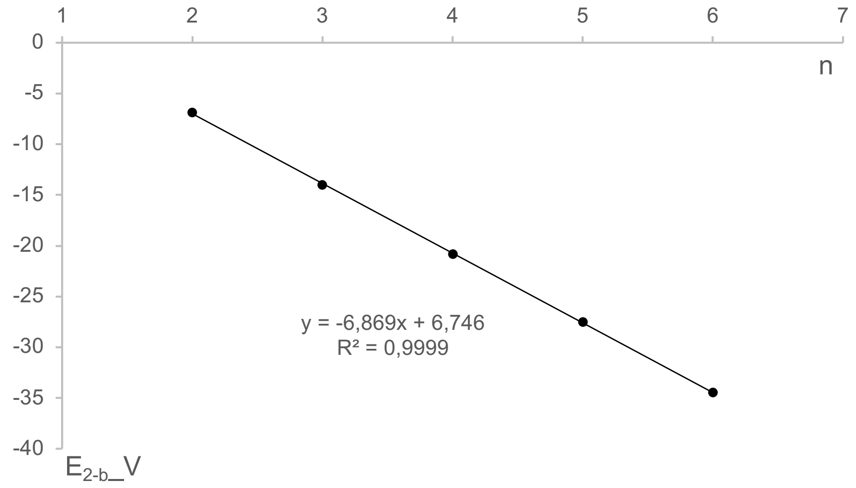

Figure S11. Linear relation between $E_{int}^{SM}[2-b]$ [kcal/mol] and number of molecules in structure V.

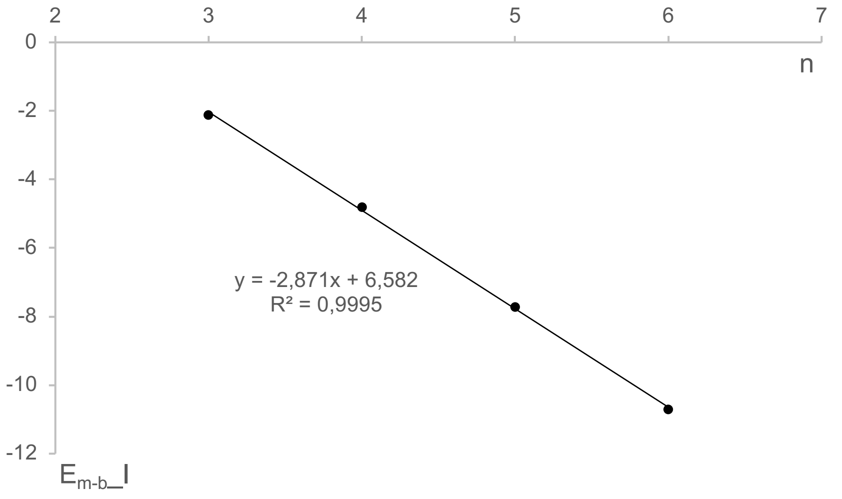

Figure S12. Linear relation between $E_{int}^{SM}[m-b]$ [kcal/mol] and number of molecules in structure I.

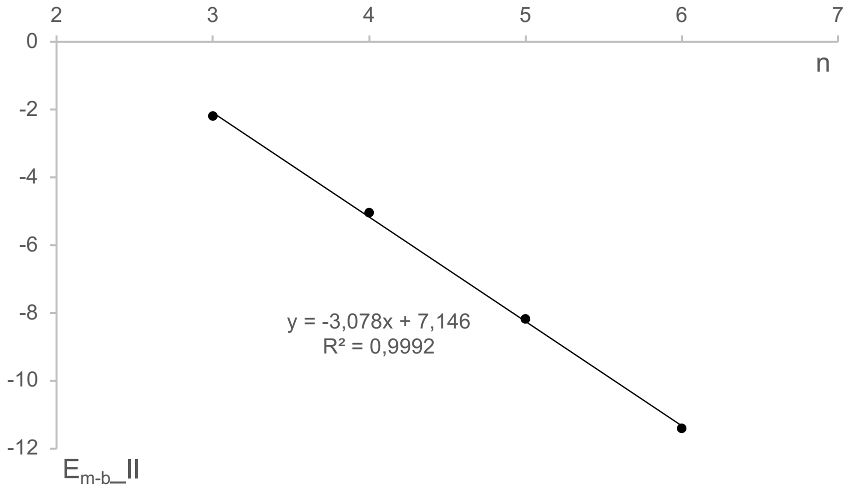

Figure S13. Linear relation between $E_{int}^{SM}[m-b]$ [kcal/mol] and number of molecules in structure II.

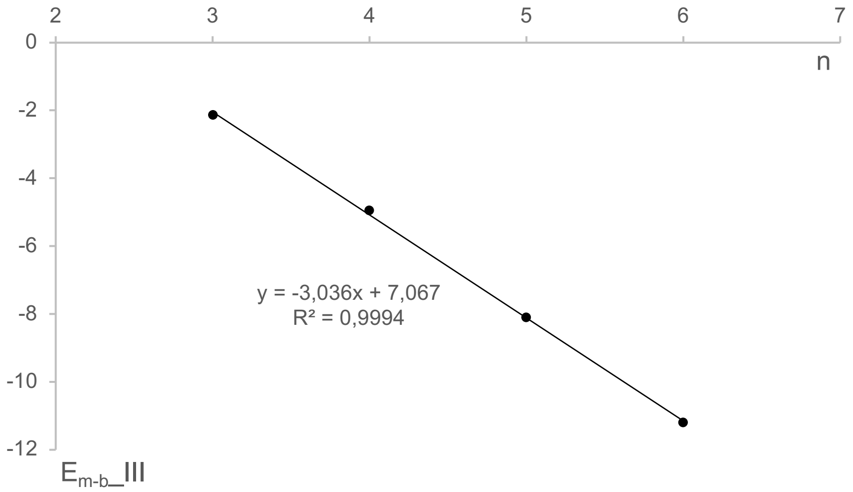

Figure S14. Linear relation between $E_{int}^{SM}[m-b]$ [kcal/mol] and number of molecules in structure III.

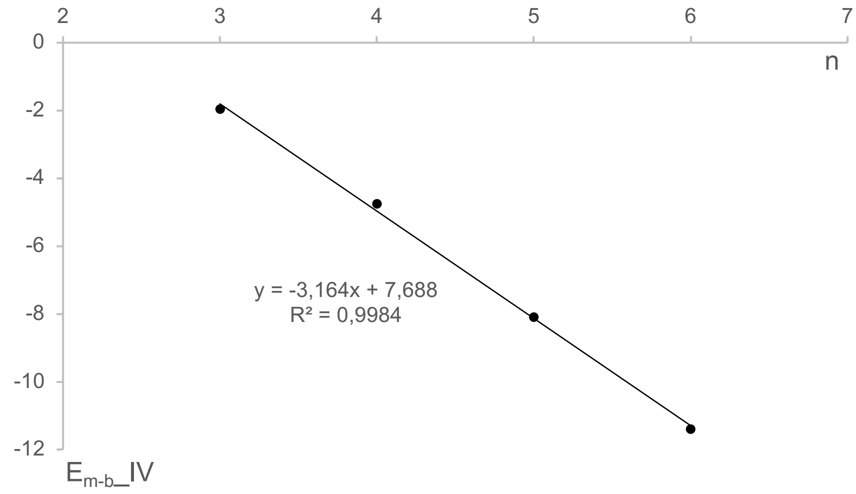

Figure S15. Linear relation between $E_{int}^{SM}[m-b]$ [kcal/mol] and number of molecules in structure IV.

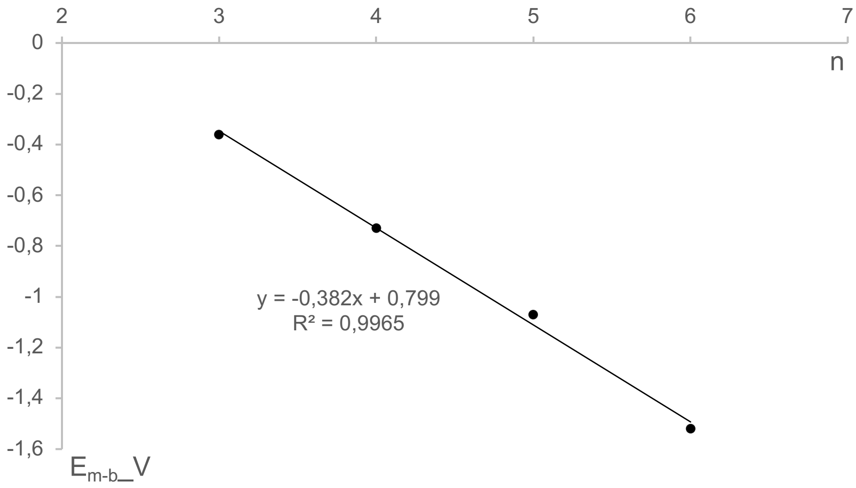

Figure S16. Linear relation between $E_{int}^{SM}[m-b]$ [kcal/mol] and number of molecules in structure V.

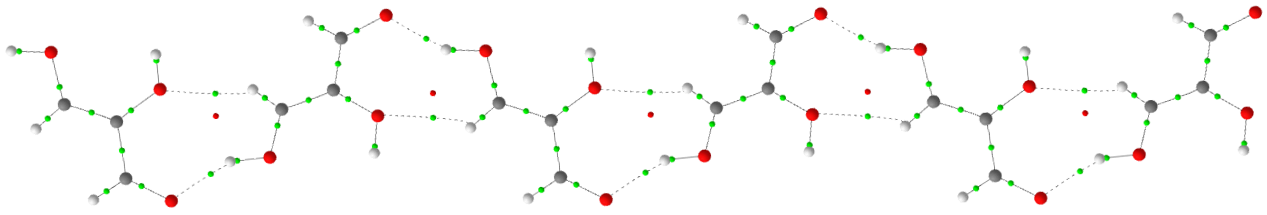

1 2 3 4 5 6

Figure S17. Molecular graph of six-molecules chain of structure III.

Table S1. Selected QTAIM parameters [au] for six-molecules chain of structure III.

	1-2		2-3		3-4		4-5		5-6	
	ρ	∇²ρ	ρ	∇²ρ	ρ	ρ	∇²ρ	ρ	∇²ρ	ρ
**$BCP_{O\text{-}H...O}$**	0.042891	0.105154	0.043044	0.103778	0.043090	0.103492	0.043044	0.103778	0.042901	0.106266
**$BCP_{C\text{-}H...O}$**	0.009358	0.037592	0.009424	0.037560	0.009440	0.037561	0.009424	0.03756	0.009448	0.037799
**RCP**	0.007591	0.032874	0.007638	0.032873	0.007647	0.032875	0.007638	0.032873	0.007625	0.033007

Table S2. Linear equation constants together with coefficient of determination to linear function, R^2, for total interaction energy and its two components, additive two-body and non-additive many-body contribution.

		a	b	R^2
**I**	E_{int}^{SM}	-16.477	20.514	0.9998
	$E_{int}^{SM}[2-b]$	-13.774	14.772	1.0000
	$E_{int}^{SM}[m-b]$	-2.871	6.582	0.9995
**II**	E_{int}^{SM}	-17.137	21.392	0.9998
	$E_{int}^{SM}[2-b]$	-14.257	15.236	1.0000
	$E_{int}^{SM}[m-b]$	-3.078	7.146	0.9992
**III**	E_{int}^{SM}	-16.937	21.780	0.9997
	$E_{int}^{SM}[2-b]$	-14.036	15.492	0.9999
	$E_{int}^{SM}[m-b]$	-3.047	7.111	0.9997
**IV**	E_{int}^{SM}	-17.885	24.080	0.9993
	$E_{int}^{SM}[2-b]$	-14.843	17.255	0.9998
	$E_{int}^{SM}[m-b]$	-3.266	8.096	0.9983
**V**	E_{int}^{SM}	-7.301	7.701	0.9999
	$E_{int}^{SM}[2-b]$	-6.928	6.943	0.9999
	$E_{int}^{SM}[m-b]$	-0.377	0.779	0.9980

Table S3. Matrix of two-body contributions to E_{int} [kcal/mol] for structure I.

n/n	1	2	3	4	5	6
**1**	-	-128928	-0.6840	-0.1814	-0.0839	-0.0518
**2**		-	-12.8928	-0.6840	-0.1814	-0.0839
**3**			-	-12.8928	-0.6840	-0.1814
**4**				-	-12.8928	-0.6840
**5**					-	-12.8928
**6**						-

Table S4. Matrix of two-body contributions to E_{int} [kcal/mol] for structure II.

n/n	1	2	3	4	5	6
**1**	-	-13.3823	-0.6977	-0.1697	-0.0681	-0.0348
**2**		-	-13.3823	-0.6976	-0.1697	-0.0681
**3**			-	-13.3823	-0.6976	-0.1697
**4**				-	-13.3823	-0.6976
**5**					-	-13.3823
**6**						-

Table S5. Matrix of two-body contributions to E_{int} [kcal/mol] for structure III.

n/n	1	2	3	4	5	6
**1**	-	-12.9160	-0.6846	-0.7208	-0.1178	-0.0862
**2**		-	-12.9159	-0.6847	-0.3068	-0.1177
**3**			-	-12.9158	-0.6846	-0.3067
**4**				-	-12.9160	-0.6847
**5**					-	-12.9160
**6**						-

Table S6. Matrix of two-body contributions to E_{int} [kcal/mol] for structure IV.

n/n	1	2	3	4	5	6
**1**	-	-13.6166	-0.6330	-0.1836	-0.1141	-0.0595
**2**		-	-13.8711	-0.7094	-0.2115	-0.1231
**3**			-	-13.7069	-0.7306	-0.2048
**4**				-	-13.6883	-0.7526
**5**					-	-13.7298
**6**						-

Table S7. Matrix of two-body contributions to E_{int} [kcal/mol] for structure V.

n/n	1	2	3	4	5	6
**1**	-	-7.1928	-0.2793	-0.0811	-0.1042	-0.0377
**2**		-	-6.4195	-0.3107	-0.1465	-0.0951
**3**			-	-6.2458	-0.3349	-0.1006
**4**				-	-6.1793	-0.3281
**5**					-	-6.4807
**6**						-

Table S8. Three-body MCI(H···OH) (au) for I, II, III, IV and V chains, being **#n** the number of monomers.

	**#n**	**1-2**	**2-3**	**3-4**	**4-5**	**5-6**
**I**						
	2	0,0016				
	3	0,0014	0,0014			
	4	0,0013	0,0011	0,0013		
	5	0,0013	0,0010	0,0011	0,0013	
	6	0,0013	0,0010	0,0010	0,0010	0,0013
**II**						
	2	0,0016				
	3	0,0013	0,0013			
	4	0,0012	0,0010	0,0013		
	5	0,0012	0,0010	0,0010	0,0012	
	6	0,0012	0,0009	0,0009	0,0009	0,0009
**III**						
	2	0,0014				
	3	0,0010	0,0011			
	4	0,0009	0,0008	0,0011		
	5	0,0008	0,0007	0,0007	0,0010	
	6	0,0008	0,0006	0,0006	0,0006	0,0010
**IV**						
	2	0,0012				
	3	0,0013	0,0011			
	4	0,0013	0,0013	0,0007		
	5	0,0008	0,0011	0,0011	0,0006	
	6	0,0005	0,0002	0,0013	0,0012	0,0007
	2	0,0031				
	3	0,0029	0,0030			
	4	0,0027	0,0040	0,0029		
	5	0,0029	0,0039	0,0040	0,0028	
	6	0,0028	0,0032	0,0040	0,0040	0,0028

Table S9. Total, σ and π contributions δ(C=C) and δ(C-C) for systems I.

n/n	1		2		3		4		5		6	
	C=C-C		C=C-C		C=C-C		C=C-C		C=C-C		C=C-C	
**Total**												
**1**	1.5255	1.0548										
**2**	1.5006	1.0818	1.5011	1.0727								
**3**	1.4965	1.0858	1.4718	1.1037	1.4963	1.0763						
**4**	1.4951	1.0871	1.4668	1.1085	1.4661	1.1082	1.4946	1.0774				
**5**	1.4946	1.0875	1.4652	1.1099	1.461	1.113	1.4644	1.1096	1.494	1.0778		
**6**	1.4944	1.0878	1.4646	1.1105	1.4594	1.1145	1.4593	1.1144	1.4637	1.1101		
σ												
**1**	0.9321	0.8786										
**2**	0.9322	0.8845	0.9324	0.8799								
**3**	0.9323	0.885	0.9327	0.8852	0.9325	0.8801						
**4**	0.9322	0.8851	0.9327	0.8857	0.9328	0.8854	0.9324	0.8801				
**5**	0.9322	0.8851	0.9328	0.8858	0.9329	0.8858	0.9328	0.8854	0.9325	0.8802		
**6**	0.9322	0.8852	0.9328	0.8859	0.9329	0.8859	0.9329	0.8858	0.9328	0.8854		
π												
**1**	0.5934	0.1762										
**2**	0.5684	0.1973	0.5687	0.1928								
**3**	0.5642	0.2008	0.5391	0.2185	0.5638	0.1962						
**4**	0.5629	0.2019	0.534	0.2228	0.5333	0.2228	0.5622	0.1972				
**5**	0.5624	0.2024	0.5325	0.2241	0.5282	0.2272	0.5316	0.2242	0.5616	0.1977		
**6**	0.5621	0.2026	0.5318	0.2247	0.5265	0.2286	0.5263	0.2286	0.5308	0.2247		

Table S10. Total, σ and π contributions δ(C=C) and δ(C-C) for systems II.

n/n	1		2		3		4		5		6	
	C=C	-C	C=C	-C	C=C	-C	C=C	-C	C=C	-C	C=C	-C
**Total**												
**1**	1.5658	1.0778										
**2**	1.5402	1.1041	1.5419	1.0960								
**3**	1.5354	1.1085	1.5116	1.1260	1.5372	1.0995						
**4**	1.5340	1.1098	1.5060	1.1311	1.5060	1.1304	1.5358	1.1006				
**5**	1.5335	1.1103	1.5043	1.1327	1.5003	1.1356	1.5043	1.1317	1.5352	1.1009		
**6**	1.5332	1.1105	1.5036	1.1332	1.4986	1.1371	1.4986	1.1370	1.5037	1.1322	1.5349	1.1011
σ												
**1**												
**2**	0.9383	0.9002	0.9397	0.8971								
**3**	0.9383	0.9006	0.9398	0.9008	0.9399	0.8972						
**4**	0.9383	0.9007	0.9398	0.9011	0.9400	0.9008	0.97	0.8973				
**5**	0.9383	0.9007	0.9398	0.9012	0.9400	0.9011	0.97	0.9008	0.9399	0.8972		
**6**	0.9383	0.9007	0.9398	0.9012	0.9401	0.9012	0.9701	0.9011	0.94	0.9008	0.94	0.8972
π												
**1**												
**2**	0.6019	0.2039	0.6022	0.1988								
**3**	0.5971	0.2079	0.5718	0.2253	0.5973	0.2023						
**4**	0.5956	0.2091	0.5662	0.2301	0.5661	0.2296	0.5958	0.2033				
**5**	0.5951	0.2095	0.5645	0.2315	0.5603	0.2345	0.5643	0.2309	0.5952	0.2037		
**6**	0.5949	0.2098	0.5638	0.2320	0.5585	0.2359	0.5585	0.2358	0.5636	0.2314	0.595	0.2039

Table S11. Total δ(C=C) and δ(C-C) for systems III.

n	C=C-C		C=C-C		C=C-C		C=C-C		C=C-C		C=C-C	
**Total**												
**1**	1.4746	1.0363										
**2**	1.4598	1.0556	1.4522	1.0569								
**3**	1.4584	1.0579	1.4333	1.0798	1.4499	1.0600						
**4**	1.4576	1.0593	1.4312	1.0832	1.4300	1.0834	1.4492	1.0620				
**5**	1.4576	1.0601	1.4295	1.0839	1.4279	1.0871	1.4285	1.0847	1.4489	1.0624		
**6**	1.4573	1.0600	1.4294	1.0846	1.4565	1.0881	1.4263	1.0883	1.4283	1.0855	1.4485	1.0622

Table S12. Total, σ and π contributions δ(C=C) and δ(C-C) for systems IV.

n	C=C-C		C=C-C		C=C-C		C=C-C		C=C-C		C=C-C	
**Total**												
**1**	1.6144	1.0569										
**2**	1.5770	1.0960	1.5813	1.0821								
**3**	1.5683	1.1041	1.5319	1.1319	1.5714	1.1090						
**4**	1.5650	1.1073	1.5195	1.1431	1.5186	1.1426	1.5671	1.1093				
**5**	1.5629	1.1099	1.5144	1.1485	1.5057	1.1537	1.5142	1.1475	1.5655	1.0937		
**6**	1.5619	1.1110	1.5107	1.1512	1.4995	1.1595	1.5018	1.1580	1.5126	1.148	1.5652	1.0942
σ												
**1**	0.9471	0.8895										
**2**	0.9437	0.8996	0.9465	0.8938								
**3**	0.9430	0.9011	0.9428	0.9045	0.9460	0.8950						
**4**	0.9428	0.9017	0.9418	0.9063	0.9421	0.9056	0.9458	0.8956				
**5**	0.9426	0.9026	0.9416	0.9078	0.9410	0.9068	0.9424	0.9067	0.9455	0.8957		
**6**	0.9425	0.9028	0.9410	0.9079	0.9407	0.9083	0.9413	0.9078	0.942	0.9066	0.9456	0.8959
π												
**1**	0.6673	0.1674										
**2**	0.6333	0.1962	0.6348	0.1883								
**3**	0.6253	0.2030	0.5892	0.2274	0.6254	0.1945						
**4**	0.6222	0.2056	0.5778	0.2368	0.5766	0.2370	0.6214	0.1971				
**5**	0.6203	0.2073	0.5728	0.2407	0.5646	0.2469	0.5718	0.2407	0.6199	0.198		
**6**	0.6193	0.2082	0.5697	0.2434	0.5588	0.2512	0.5605	0.2501	0.5706	0.2415	0.6196	0.1983

Table S13. Total, σ and π contributions δ(C=C) and δ(C-C) for systems V.

	**C=C-C**		**C=C-C**		**C=C-C**		**C=C-C**		**C=C-C**		**C=C-C**	
**Total**												
**1**	0.9595	0.9515										
**2**	0.9575	0.9616	0.9570	0.9534								
**3**	0.9573	0.9623	0.9548	0.9635	0.9567	0.9544						
**4**	0.9570	0.963	0.9546	0.9635	0.9546	0.9649	0.9563	0.9546				
**5**	0.9571	0.963	0.9542	0.9641	0.9544	0.9645	0.9541	0.9648	0.9563	0.9549		
**6**	0.9570	0.9632	0.9542	0.9645	0.9538	0.9647	0.9542	0.9641	0.9541	0.9645	0.9565	0.9544
**σ**												
**1**												
**2**	0.8966	0.8868	0.8964	0.8825								
**3**	0.8964	0.8871	0.8942	0.8878	0.8961	0.8831						
**4**	0.8962	0.8876	0.8941	0.8879	0.8939	0.8885	0.8958	0.8958				
**5**	0.8963	0.8876	0.8937	0.8881	0.8938	0.8883	0.8935	0.8935	0.8957	0.8834		
**6**	0.8962	0.8876	0.8937	0.8884	0.8933	0.8885	0.8935	0.8880	0.8934	0.8883	0.8960	0.8832
**π**												
**1**												
**2**	0.0609	0.0748	0.0606	0.0709								
**3**	0.0609	0.0752	0.0606	0.0757	0.0606	0.0713						
**4**	0.0608	0.0754	0.0605	0.0756	0.0606	0.0763	0.0605	0.0713				
**5**	0.0608	0.0754	0.0605	0.0760	0.0606	0.0762	0.0606	0.0764	0.0605	0.0714		
**6**	0.0608	0.0756	0.0605	0.0761	0.0605	0.0762	0.0607	0.0761	0.0607	0.0762	0.0605	0.0713

Table S14. Delocalization indices for HB formation: Total, σ and π contributions δ(O··H) and δ(HO) for systems.

	HO	O···HO		O···HO		O···HO		O···HO		O···HO	
**Total**											
**1**	0.6245										
**2**	0.6132	0.0939	0.4523								
**3**	0.6104	0.0945	0.4416	0.0949	0.4444						
**4**	0.6094	0.0947	0.4389	0.0957	0.4336	0.0952	0.4423				
**5**	0.609	0.0948	0.438	0.0959	0.431	0.0959	0.4315	0.0953	0.4416		
**6**	0.6088	0.0948	0.4376	0.0959	0.43	0.0961	0.4289	0.096	0.4307	0.0954	0.4412
σ											
**1**	0.6027										
**2**	0.5927	0.0905	0.4376								
**3**	0.5902	0.0911	0.4278	0.0914	0.4302						
**4**	0.5893	0.0914	0.4254	0.0922	0.4204	0.0917	0.4283				
**5**	0.5889	0.0914	0.4246	0.0924	0.418	0.0924	0.4184	0.0918	0.4276		
**6**	0.5888	0.0915	0.4242	0.0924	0.4171	0.0926	0.416	0.0925	0.4177	0.0918	0.4273
π											
**1**	0.0218										
**2**	0.0205	0.0034	0.0147								
**3**	0.0202	0.0034	0.0138	0.0035	0.0142						
**4**	0.0201	0.0034	0.0135	0.0034	0.0132	0.0035	0.014				
**5**	0.0201	0.0034	0.0135	0.0035	0.013	0.0035	0.0131	0.0035	0.014		
**6**	0.0201	0.0034	0.0134	0.0035	0.013	0.0035	0.0129	0.0035	0.013	0.0035	0.0139

Table S15. Delocalization indices for HB formation: δ(O··H) and δ(HO) for systems II being #n the number of monomers. Total, σ and π contributions.

	HO	O···HO		O···HO		O···HO		O···HO		O···HO	
**Total**											
**1**	0.6310										
**2**	0.6197	0.0990	0.4603								
**3**	0.6170	0.0999	0.4497	0.1004	0.4523						
**4**	0.6161	0.1001	0.4473	0.1014	0.4417	0.1008	0.4504				
**5**	0.6158	0.1001	0.4463	0.1016	0.4392	0.1017	0.4396	0.1009	0.4495		
**6**	0.6156	0.1001	0.4460	0.1017	0.4384	0.1019	0.4372	0.1018	0.4389	0.1009	0.4492
σ											
**1**											
**2**	0.5985	0.0955	0.4448								
**3**	0.5961	0.0963	0.4352	0.0968	0.4374						
**4**	0.5954	0.0966	0.433	0.0978	0.4278	0.0972	0.4356				
**5**	0.595	0.0966	0.4322	0.098	0.4255	0.098	0.4258	0.0972	0.4349		
**6**	0.5949	0.0966	0.4318	0.0981	0.4247	0.0983	0.4258	0.0982	0.4252	0.0973	0.4346
π											
**1**											
**2**	0.0211	0.0035	0.0155								
**3**	0.0209	0.0035	0.0145	0.0036	0.0149						
**4**	0.0208	0.0035	0.0143	0.0036	0.014	0.0036	0.0148				
**5**	0.0207	0.0035	0.0142	0.0036	0.137	0.0036	0.0138	0.0036	0.0147		
**6**	0.0207	0.0035	0.0141	0.0036	0.136	0.0036	0.0136	0.0036	0.0137	0.0036	0.0147

Table S16. Delocalization indices for HB formation: δ(O··H) and δ(HO) for systems III being #n the number of monomers.

	HO	O···HO		O···HO		O···HO		O···HO		O···HO	
**Total**											
**1**	0.6349										
**2**	0.6223	0.0925	0.4653								
**3**	0.6189	0.0937	0.4536	0.0934	0.4582						
**4**	0.6176	0.0941	0.4506	0.0948	0.4463	0.0940	0.4558				
**5**	0.617	0.0941	0.4493	0.0952	0.4434	0.0952	0.444	0.0941	0.4549		
**6**	0.6167	0.0943	0.4489	0.0953	0.4421	0.0957	0.441	0.0954	0.4431	0.0942	0.4545

Table S17. Delocalization indices for HB formation: δ(O··H) and δ(HO) for systems IV being #n the number of monomers. Total, σ and π contributions.

	**HO**	**O···HO**		**O···HO**		**O···HO**		**O···HO**		**O···HO**	
**Total**											
**1**	0.6161										
**2**	0.6048	0.0957	0.4659								
**3**	0.6011	0.0968	0.4548	0.1005	0.4526						
**4**	0.6002	0.0993	0.4469	0.1017	0.4398	0.1064	0.4426				
**5**	0.599	0.1058	0.4373	0.1042	0.4343	0.1052	0.4333	0.1079	0.4409		
**6**	0.5986	0.1091	0.4320	0.1145	0.4208	0.1039	0.432	0.1043	0.4336	0.1063	0.442
σ											
**1**	0.5938										
**2**	0.584	0.0905	0.0923								
**3**	0.5807	0.0911	0.0934	0.0967	0.4375						
**4**	0.5799	0.0914	0.0958	0.098	0.4259	0.1024	0.4278				
**5**	0.5789	0.0914	0.102	0.1004	0.4207	0.1013	0.4197	0.1038	0.4262		
**6**	0.5785	0.0915	0.1052	0.1103	0.4076	0.1000	0.4187	0.1004	0.42	0.1023	0.4273
π											
**1**	0.0223										
**2**	0.0208	0.0034	0.0158								
**3**	0.0204	0.0035	0.0147	0.0038	0.0151						
**4**	0.0203	0.0036	0.0142	0.0037	0.0138	0.0039	0.0148				
**5**	0.0202	0.0038	0.014	0.0038	0.0135	0.0039	0.0138	0.0041	0.1470		
**6**	0.0201	0.0039	0.0138	0.0043	0.0133	0.0039	0.0133	0.0039	0.1360	0.004	0.0147

Table S18. Delocalization indices for HB formation: δ(O··H) and δ(HO) for systems V being #n the number of monomers. Total, σ and π contributions.

	**HO**	**O···HO**		**O···HO**		**O···HO**		**O···HO**		**O···HO**	
**Total**											
**1**	0.6577										
**2**	0.6535	0.0701	0.55								
**3**	0.6522	0.072	0.5425	0.0722	0.5444						
**4**	0.6517	0.0741	0.5391	0.0594	0.5556	0.0739	0.5397				
**5**	0.6516	0.0735	0.5394	0.0626	0.5502	0.0620	0.5499	0.0741	0.5395		
**6**	0.6521	0.0732	0.5395	0.0701	0.5404	0.0579	0.5581	0.0624	0.5499	0.0728	0.5423
σ											
**1**											
**2**	0.6261	0.0678	0.5279								
**3**	0.6249	0.0697	0.5209	0.0698	0.5225						
**4**	0.6245	0.0717	0.5176	0.0575	0.5337	0.0715	0.5181				
**5**	0.6244	0.0711	0.5179	0.0606	0.5286	0.0600	0.5283	0.0717	0.5179		
**6**	0.6249	0.0708	0.5181	0.0678	0.5191	0.0560	0.5362	0.0603	0.5283	0.0704	0.5207
π											
**1**											
**2**	0.0274	0.0023	0.0222								
**3**	0.0273	0.0023	0.0216	0.0024	0.0219						
**4**	0.0272	0.0024	0.0215	0.0019	0.0219	0.0024	0.0216				
**5**	0.0272	0.0024	0.0215	0.0021	0.0217	0.0021	0.0216	0.0025	0.0216		
**6**	**0.0272**	0.0024	0.0214	0.0023	0.0213	0.0019	0.0219	0.0021	0.0216	0.0024	0.0216

Table S19. NPA charges for the atoms involved in the RAHB for each monomer

n	HO			O···HO			O···HO			O···HO			O···HO			O···HO	
I																	
**1**	0.485	-0.631	-0.553														
**2**	0.490	-0.608	-0.608	0.504	-0.662	-0.578											
**3**	0.492	-0.618	-0.618	0.508	-0.647	-0.634	0.507	-0.662									
**4**	0.492	-0.614	-0.621	0.509	-0.644	-0.644	0.511	-0.646	-0.639	0.508	-0.662	-0.586					
**5**	0.492	-0.614	-0.622	0.510	-0.643	-0.646	0.513	-0.643	-0.649	0.512	-0.646	-0.641	0.508	-0.662	-0.586		
**6**	0.493	-0.614	-0.622	0.510	-0.643	-0.647	0.513	-0.643	-0.652	0.513	-0.643	-0.651	0.513	-0.646	-0.642	0.508	-0.662
II																	
**1**	0.488	-0.617	-0.529														
**2**	0.494	-0.605	-0.593	0.510	-0.645	-0.554											
**3**	0.496	-0.602	-0.602	0.515	-0.630	-0.617	0.513	-0.644	-0.559								
**4**	0.496	-0.602	-0.605	0.516	-0.628	-0.627	0.518	-0.629	-0.623	0.514	-0.643	-0.561					
**5**	0.496	-0.601	-0.606	0.516	-0.627	-0.630	0.519	-0.626	-0.632	0.519	-0.628	-0.625	0.514	-0.643	-0.562		
**6**	0.496	-0.601	-0.606	0.516	-0.627	-0.631	0.520	-0.625	-0.635	0.520	-0.625	-0.634	0.519	-0.628	-0.625	0.515	-0.643
III																	
**1**	0.485	-0.648	-0.533														
**2**	0.491	-0.635	-0.567	0.507	-0.667	-0.560											
**3**	0.496	-0.634	-0.573	0.512	-0.652	-0.675	0.483										
**4**	0.494	-0.633	-0.575	0.513	-0.650	-0.600	0.515	-0.653	-0.601	0.511	-0.668	-0.569					
**5**	0.494	-0.633	-0.576	0.514	-0.649	-0.603	0.517	-0.651	-0.607	0.516	-0.652	-0.603	0.512	-0.668	-0.570		
**6**	0.494	-0.633	-0.576	0.514	-0.649	-0.604	0.517	-0.650	-0.609	0.517	-0.651	-0.609	0.517	-0.652	-0.604	0.512	-0.668
IV																	
**1**	0.485	-0.651	-0.541														
**2**	0.491	-0.635	-0.599	0.505	-0.674	-0.565											
**3**	0.492	-0.632	-0.615	0.511	-0.653	-0.629	0.508	-0.673	-0.573								
**4**	0.493	-0.63	-0.619	0.512	-0.649	-0.647	0.515	-0.652	-0.634	0.508	-0.674	-0.576					
**5**	0.493	-0.629	-0.615	0.51	-0.65	-0.652	0.516	-0.647	-0.655	0.515	-0.651	-0.637	0.508	-0.674	-0.577		
**6**	0.494	-0.629	-0.613	0.51	-0.65	-0.644	0.512	-0.648	-0.663	0.518	-0.647	-0.66	0.517	-0.639	0.509	-0.673	-0.577
V																	
**1**	0.467	-0.726	-0.517														
**2**	0.47	-0.722	-0.554	0.488	-0.75	-0.525											
**3**	0.47	-0.722	-0.558	0.49	-0.747	-0.563	0.489	-0.75	-0.527								
**4**	0.471	-0.723	-0.558	0.49	-0.748	-0.573	0.495	-0.744	-0.565	0.491	-0.752	-0.528					
**5**	0.471	-0.722	-0.559	0.491	-0.748	-0.574	0.496	0.48	-0.575	0.496	-0.745	-0.565	0.491	-0.751	-0.528		
**6**	0.47	-0.722	-0.559	0.491	-0.748	-0.57	0.493	-0.747	-0.576	0.496	-0.743	-0.576	0.496	-0.745	-0.565	0.49	-0.751

Table S20. Atom coordinates of examined complexes corresponding to single point geometries at ωB97XD/aug-cc-pVTZ level of theory (for structures I-III) for a chain of 6 molecules.

Structure I				Structure II				Structure III			
O	3.157300	1.558878	5.015023	Br	3.157300	4.374378	3.564523	C	0.918217	0.675776	0.364304
O	3.157300	3.281478	0.616523	O	3.157300	1.558878	5.015023	C	2.265517	0.256076	0.270504
H	3.157300	2.843022	-0.259923	O	3.157300	3.281478	0.616523	C	2.718617	-0.060224	-0.962796
C	3.157300	1.507678	3.784123	H	3.157300	2.843022	-0.259923	H	0.442017	0.675176	-0.463996
H	3.157300	0.643078	3.391523	C	3.157300	1.507678	3.784123	H	3.879217	0.301276	1.280004
C	3.157300	2.625578	2.885923	H	3.157300	0.643078	3.391523	H	4.030083	-0.611376	-2.129004
C	3.157300	2.360578	1.556223	C	3.157300	2.625578	2.885923	H	2.170617	0.010276	-1.710496
H	3.157300	1.450178	1.285023	C	3.157300	2.360578	1.556223	O	0.358417	1.043176	1.409004
O	3.157300	1.558878	-1.372177	H	3.157300	1.450178	1.285023	O	2.988117	0.190376	1.409804
O	3.157300	3.281478	-5.770677	Br	3.157300	4.374378	-2.822677	O	3.961417	-0.462624	-1.162796
H	3.157300	2.843022	-6.647123	O	3.157300	1.558878	-1.372177	C	4.382283	-1.147724	-4.639696
C	3.157300	1.507678	-2.603077	O	3.157300	3.281478	-5.770677	C	3.034983	-1.567424	-4.733496
H	3.157300	0.643078	-2.995677	H	3.157300	2.843022	-6.647123	C	2.581883	-1.883724	-5.966796
C	3.157300	2.625578	-3.501277	C	3.157300	1.507678	-2.603077	H	4.858483	-1.148324	-5.467996
C	3.157300	2.360578	-4.830977	H	3.157300	0.643078	-2.995677	H	1.421283	-1.522224	-3.723996
H	3.157300	1.450178	-5.102177	C	3.157300	2.625578	-3.501277	H	1.270417	-2.434876	-7.133004
O	3.157300	1.558878	11.402223	C	3.157300	2.360578	-4.830977	H	3.129883	-1.813224	-6.714496
O	3.157300	3.281478	7.003723	H	3.157300	1.450178	-5.102177	O	4.942083	-0.780324	-3.594996
H	3.157300	2.843022	6.127277	Br	3.157300	4.374378	9.951723	O	2.312383	-1.633124	-3.594196
C	3.157300	1.507678	10.171323	O	3.157300	1.558878	11.402223	O	1.339083	-2.286124	-6.166796
H	3.157300	0.643078	9.778723	O	3.157300	3.281478	7.003723	C	0.918217	-2.971224	-9.643696
C	3.157300	2.625578	9.273123	H	3.157300	2.843022	6.127277	C	2.265517	-3.390924	-9.737496
C	3.157300	2.360578	7.943423	C	3.157300	1.507678	10.171323	C	2.718617	-3.707224	-10.970796
H	3.157300	1.450178	7.672223	H	3.157300	0.643078	9.778723	H	0.442017	-2.971824	-10.471996
O	3.157300	1.558878	-7.759377	C	3.157300	2.625578	9.273123	H	3.879217	-3.345724	-8.727996
O	3.157300	3.281478	-12.157877	C	3.157300	2.360578	7.943423	H	4.030083	-4.258376	-12.137004
H	3.157300	2.843022	-13.034323	H	3.157300	1.450178	7.672223	H	2.170617	-3.636724	-11.718496
C	3.157300	1.507678	-8.990277	Br	3.157300	4.374378	-9.209877	O	0.358417	-2.603824	-8.598996
H	3.157300	0.643078	-9.382877	O	3.157300	1.558878	-7.759377	O	2.988117	-3.456624	-8.598196
C	3.157300	2.625578	-9.888477	O	3.157300	3.281478	-12.157877	O	3.961417	-4.109624	-11.170796
C	3.157300	2.360578	-11.218177	H	3.157300	2.843022	-13.034323	C	4.382283	2.499276	5.368304
H	3.157300	1.450178	-11.489377	C	3.157300	1.507678	-8.990277	C	3.034983	2.079576	5.274504
O	3.157300	1.558878	17.789423	H	3.157300	0.643078	-9.382877	C	2.581883	1.763276	4.041204
O	3.157300	3.281478	13.390923	C	3.157300	2.625578	-9.888477	H	4.858483	2.498676	4.540004
H	3.157300	2.843022	12.514477	C	3.157300	2.360578	-11.218177	H	1.421283	2.124776	6.284004
C	3.157300	1.507678	16.558523	H	3.157300	1.450178	-11.489377	H	1.270417	1.212124	2.874996
H	3.157300	0.643078	16.165923	Br	3.157300	4.374378	16.338923	H	3.129883	1.833776	3.293504
C	3.157300	2.625578	15.660323	O	3.157300	1.558878	17.789423	O	4.942083	2.866676	6.413004
C	3.157300	2.360578	14.330623	O	3.157300	3.281478	13.390923	O	2.312383	2.013876	6.413804
H	3.157300	1.450178	14.059423	H	3.157300	2.843022	12.514477	O	1.339083	1.360876	3.841204
O	3.157300	1.558878	24.176623	C	3.157300	1.507678	16.558523	C	0.918217	4.322776	10.372304
O	3.157300	3.281478	19.778123	H	3.157300	0.643078	16.165923	C	2.265517	3.903076	10.278504
H	3.157300	2.843022	18.901677	C	3.157300	2.625578	15.660323	C	2.718617	3.586776	9.045204
C	3.157300	1.507678	22.945723	C	3.157300	2.360578	14.330623	H	0.442017	4.322176	9.544004
H	3.157300	0.643078	22.553123	H	3.157300	1.450178	14.059423	H	3.879217	3.948276	11.288004
C	3.157300	2.625578	22.047523	Br	3.157300	4.374378	22.726123	H	4.030083	3.035624	7.878996
C	3.157300	2.360578	20.717823	O	3.157300	1.558878	24.176623	H	2.170617	3.657276	8.297504
H	3.157300	1.450178	20.446623	O	3.157300	3.281478	19.778123	O	0.358417	4.690176	11.417004
H	3.157300	3.623110	-9.501398	H	3.157300	2.843022	18.901677	O	2.988117	3.837376	11.417804
H	3.157300	3.623110	-3.114198	C	3.157300	1.507678	22.945723	O	3.961417	3.184376	8.845204
H	3.157300	3.623110	3.273002	H	3.157300	0.643078	22.553123	C	4.382283	6.146276	15.376304
H	3.157300	3.623110	9.660202	C	3.157300	2.625578	22.047523	C	3.034983	5.726576	15.282504
H	3.157300	3.623110	16.047402	C	3.157300	2.360578	20.717823	C	2.581883	5.410276	14.049204

H	3.157300	3.623110	22.434602	H	3.157300	1.450178	20.446623	H	4.858483	6.145676	14.548004
								H	1.421283	5.771776	16.292004
								H	1.270417	4.859124	12.882996
								H	3.129883	5.480776	13.301504
								O	4.942083	6.513676	16.421004
								O	2.312383	5.660876	16.421804
								O	1.339083	5.007876	13.849204

Table S21. Atom coordinates of examined complexes corresponding to optimized geometries at ωB97XD/6-31++G(d.p) level of theory (for structures IV-V).

Structure IV

1 MOLECULE

O	-2.365624	0.543977	0.000000
O	2.308288	-0.078910	0.000000
H	2.909973	-0.827963	0.000000
C	-1.363267	-0.143462	0.000000
H	-1.450182	-1.252955	0.000000
C	0.000000	0.375558	0.000000
C	1.027206	-0.486277	0.000000
H	0.859353	-1.563622	0.000000
H	0.155909	1.449093	0.000000

2 MOLECULES

O	0.522377	-1.454518	0.000000
O	-3.578355	-3.767861	0.000000
H	-3.861722	-4.686906	0.000000
C	-0.173370	-2.463064	0.000000
H	0.326043	-3.451365	0.000000
C	-1.618759	-2.476814	0.000000
C	-2.247065	-3.666628	0.000000
H	-1.683209	-4.600496	0.000000
O	2.824836	4.857985	0.000000
O	-0.131543	1.174604	0.000000
H	0.000000	0.203523	0.000000
C	2.550685	3.670441	0.000000
H	3.369518	2.914875	0.000000
C	1.208126	3.117753	0.000000
C	1.053403	1.779791	0.000000
H	1.923958	1.120162	0.000000
H	0.357785	3.791263	0.000000
H	-2.169022	-1.541610	0.000000

3 MOLECULES

O	1.118112	-4.141259	0.000000
O	-0.961468	-8.362020	0.000000
H	-0.682228	-9.282511	0.000000
C	1.111572	-5.367943	0.000000
H	2.081765	-5.899935	0.000000
C	-0.071564	-6.193475	0.000000
C	0.081030	-7.531515	0.000000
H	1.072292	-7.985612	0.000000

Structure V

1 MOLECULE

O	-2.385062	0.712436	0.000000
O	2.294993	-0.150036	0.000000
H	2.950633	-0.852083	0.000000
C	-1.436689	-0.036730	0.000000
H	-1.602971	-1.137188	0.000000
C	0.000000	0.428341	0.000000
C	0.998983	-0.716539	0.000000
H	0.844420	-1.346672	0.888914
H	0.155143	1.066491	-0.877279
H	0.155143	1.066491	0.877279
H	0.844420	-1.346672	-0.888914

2 MOLECULES

O	-0.345675	-1.586732	0.000000
O	4.080060	-3.330124	0.000000
H	4.586796	-4.146394	0.000000
C	0.450488	-2.502154	0.000000
H	0.069534	-3.544127	0.000000
C	1.942962	-2.316823	0.000000
C	2.701330	-3.634784	0.000000
H	2.425023	-4.222118	0.889093
O	-3.397665	4.701491	0.000000
O	-0.078851	1.275700	0.000000
H	0.000000	0.310872	0.000000
C	-3.046762	3.544002	0.000000
H	-3.813416	2.737062	0.000000
C	-1.605151	3.098417	0.000000
C	-1.453656	1.586272	0.000000
H	-1.951967	1.163547	0.886500
H	-1.111194	3.532234	-0.877170
H	2.217577	-1.718498	-0.876946
H	-1.111194	3.532234	0.877170
H	-1.951967	1.163547	-0.886500
H	2.217577	-1.718498	0.876946
H	2.425023	-4.222118	-0.889093

3 MOLECULES

O	4.661219	-0.785448	0.000000
O	6.292848	-5.250600	0.000000

O	0.959481	2.382280	0.000000
O	-0.578880	-2.077867	0.000000
H	-0.113261	-2.943556	0.000000
C	1.076524	1.157684	0.000000
H	2.098231	0.730259	0.000000
C	0.000000	0.206953	0.000000
C	0.317125	-1.108800	0.000000
H	1.362095	-1.426086	0.000000
O	-0.554009	8.817609	0.000000
O	-1.069353	4.120991	0.000000
H	-0.427299	3.375723	0.000000
C	-0.153432	7.665231	0.000000
H	0.942280	7.464340	0.000000
C	-0.990220	6.480961	0.000000
C	-0.400903	5.268010	0.000000
H	0.687791	5.180579	0.000000
H	-2.069405	6.592532	0.000000
H	-1.030281	0.546849	0.000000
H	-1.053846	-5.733079	0.000000

4 MOLECULES

O	3.582725	-6.521841	0.000000
O	7.815503	-8.580306	0.000000
H	8.158518	-9.479333	0.000000
C	4.343046	-7.485359	0.000000
H	3.903758	-8.500877	0.000000
C	5.783413	-7.410380	0.000000
C	6.483202	-8.562285	0.000000
H	5.976554	-9.528407	0.000000
O	-0.295031	-1.263682	0.000000
O	3.639671	-3.863695	0.000000
H	3.800350	-4.835338	0.000000
C	0.363211	-2.304874	0.000000
H	-0.186854	-3.265167	0.000000
C	1.792756	-2.401585	0.000000
C	2.341705	-3.640641	0.000000
H	1.704496	-4.527807	0.000000
O	-4.151133	3.896719	0.000000
O	-0.171898	1.363327	0.000000
H	0.000000	0.391036	0.000000
C	-3.478237	2.864495	0.000000
H	-4.012983	1.895045	0.000000
C	-2.046654	2.791184	0.000000
C	-1.473074	1.562981	0.000000
H	-2.091275	0.662409	0.000000
O	-6.412921	10.150949	0.000000
O	-3.464491	6.461144	0.000000
H	-3.601850	5.483762	0.000000
C	-6.136426	8.962264	0.000000
H	-6.955677	8.207530	0.000000
C	-4.797381	8.408689	0.000000
C	-4.643043	7.068321	0.000000
H	-5.515843	6.411070	0.000000
H	-3.945426	9.080449	0.000000
H	-1.452156	3.698762	0.000000
H	2.404566	-1.505833	0.000000
H	6.279298	-6.445089	0.000000

H	7.093075	-5.781891	0.000000
C	5.560014	-1.600881	0.000000
H	6.609604	-1.241228	0.000000
C	5.338977	-3.087893	0.000000
C	6.636192	-3.881159	0.000000
H	7.230230	-3.621399	0.889492
O	-1.580776	2.368917	0.000000
O	1.806655	-0.983157	0.000000
H	2.771030	-1.076080	0.000000
C	-0.425276	1.996599	0.000000
H	0.380918	2.758995	0.000000
C	0.000000	0.555179	0.000000
C	1.512420	0.392099	0.000000
H	1.937028	0.888512	0.886776
O	-8.503691	2.946295	0.000000
O	-4.120948	1.068891	0.000000
H	-3.191100	1.340741	0.000000
C	-7.296406	3.029194	0.000000
H	-6.813578	4.032133	0.000000
C	-6.367177	1.841390	0.000000
C	-4.901135	2.241162	0.000000
H	-4.685213	2.858256	0.886447
H	-6.595073	1.224963	0.877310
H	-0.439389	0.064205	0.876727
H	4.733388	-3.347528	0.876681
H	-6.595073	1.224963	-0.877310
H	-4.685213	2.858256	-0.886447
H	-0.439389	0.064205	-0.876727
H	1.937028	0.888512	-0.886776
H	4.733388	-3.347528	-0.876681
H	7.230230	-3.621399	-0.889492

4 MOLECULES

O	-2.577618	-7.649045	0.000000
O	-7.139678	-8.978670	0.000000
H	-7.730600	-9.736375	0.000000
C	-3.450052	-8.493272	0.000000
H	-3.159185	-9.564057	0.000000
C	-4.920140	-8.176879	0.000000
C	-5.798014	-9.418801	0.000000
H	-5.579808	-10.029220	0.889323
O	0.555832	-1.370356	0.000000
O	-2.754379	-4.798577	0.000000
H	-2.836205	-5.764458	0.000000
C	0.196899	-2.529826	0.000000
H	0.968630	-3.327583	0.000000
C	-1.238370	-2.974399	0.000000
C	-1.382915	-4.488909	0.000000
H	-0.881651	-4.907897	-0.886785
O	3.796895	4.560355	0.000000
O	0.169538	1.468440	0.000000
H	0.000000	0.516316	0.000000
C	3.324351	3.441388	0.000000
H	4.014392	2.572225	0.000000
C	1.852666	3.140467	0.000000
C	1.564312	1.646678	0.000000
H	2.023697	1.181271	0.885958
O	4.510669	11.559215	0.000000

5 MOLECULES

O	3.232803	-10.210855	0.000000
O	7.257973	-12.643049	0.000000
H	7.524304	-13.567435	0.000000
C	3.898202	-11.243428	0.000000
H	3.364169	-12.211591	0.000000
C	5.337843	-11.302317	0.000000
C	5.932980	-12.511163	0.000000
H	5.345456	-13.429766	0.000000
O	0.669090	-4.123985	0.000000
O	3.819510	-7.630078	0.000000
H	3.739112	-8.614879	0.000000
C	1.041123	-5.299378	0.000000
H	0.264127	-6.086946	0.000000
C	2.398131	-5.754440	0.000000
C	2.619202	-7.092275	0.000000
H	1.780451	-7.792150	0.000000
O	-1.781369	1.800105	0.000000
O	1.459349	-1.627703	0.000000
H	1.379703	-2.613960	0.000000
C	-1.368777	0.637507	0.000000
H	-2.119985	-0.174891	0.000000
C	0.000000	0.223418	0.000000
C	0.251943	-1.110448	0.000000
H	-0.570958	-1.828625	0.000000
O	-4.362842	7.688061	0.000000
O	-1.065024	4.315482	0.000000
H	-1.122064	3.327546	0.000000
C	-3.940225	6.529837	0.000000
H	-4.681188	5.707335	0.000000
C	-2.563646	6.133986	0.000000
C	-2.284920	4.806590	0.000000
H	-3.091750	4.070362	0.000000
O	-5.091323	14.290244	0.000000
O	-3.089886	10.012038	0.000000
H	-3.453327	9.093397	0.000000
C	-5.099886	13.069648	0.000000
H	-6.073281	12.527800	0.000000
C	-3.927829	12.218151	0.000000
C	-4.092608	10.878732	0.000000
H	-5.094992	10.444441	0.000000
H	-2.942023	12.670835	0.000000
H	-1.777718	6.881747	0.000000
H	0.798769	0.957050	0.000000
H	3.216494	-5.042639	0.000000
H	5.919264	-10.386233	0.000000

6 MOLECULES

O	6.291279	-4.102071	0.000000
O	10.990778	-3.764950	0.000000
H	11.728227	-4.425678	0.000000
C	7.446887	-4.536373	0.000000
H	7.585342	-5.633417	0.000000
C	8.639346	-3.746173	0.000000
C	9.833880	-4.388990	0.000000
H	9.875669	-5.480846	0.000000
O	2.645541	7.172557	0.000000
H	2.907593	6.239306	0.000000
C	4.600991	10.352518	0.000000
H	5.607202	9.876488	0.000000
C	3.418447	9.416588	0.000000
C	3.819425	7.949900	0.000000
H	4.436713	7.734018	-0.886613
H	2.800550	9.641890	0.877078
H	1.399918	3.619554	0.876530
H	-1.734825	-2.542139	0.877019
H	-5.140515	-7.555445	0.876327
H	4.436713	7.734018	0.886613
H	2.800550	9.641890	-0.877078
H	1.399918	3.619554	-0.876530
H	2.023697	1.181271	-0.885958
H	-0.881651	-4.907897	0.886785
H	-1.734825	-2.542139	-0.877019
H	-5.579808	-10.029220	-0.889323
H	-5.140515	-7.555445	-0.876327

5 MOLECULES

O	1.073201	-11.341112	0.000000
O	-2.193186	-14.793212	0.000000
H	-2.317746	-15.745757	0.000000
C	0.745650	-12.510021	0.000000
H	1.537550	-13.286599	0.000000
C	-0.682157	-12.980277	0.000000
C	-0.813274	-14.495655	0.000000
H	-0.316852	-14.912835	0.889109
O	1.073201	-4.350674	0.000000
O	-0.378039	-8.888341	0.000000
H	-0.027834	-9.791993	0.000000
C	1.266573	-5.549271	0.000000
H	2.312334	-5.920528	0.000000
C	0.177785	-6.583917	0.000000
C	0.717212	-8.006701	0.000000
H	1.351813	-8.161729	-0.886982
O	0.935732	2.426318	0.000000
O	-0.640777	-2.071792	0.000000
H	-0.307195	-2.980184	0.000000
C	1.089480	1.222113	0.000000
H	2.123024	0.817109	0.000000
C	-0.029466	0.219872	0.000000
C	0.474356	-1.215690	0.000000
H	1.105503	-1.386592	0.886219
O	1.226953	9.221008	0.000000
O	-0.611321	4.824596	0.000000
H	-0.335419	3.897227	0.000000
C	1.312378	8.009306	0.000000
H	2.320889	7.546009	0.000000
C	0.136596	7.074699	0.000000
C	0.553262	5.611535	0.000000
H	1.173196	5.403341	0.886267
O	-1.500480	15.719426	0.000000
O	-1.031538	10.975160	0.000000
H	-0.352671	10.283161	0.000000
C	-0.845141	14.702319	0.000000
H	0.266344	14.763953	0.000000

O	12.745622	-5.737629	0.000000
O	16.966797	-3.662944	0.000000
H	17.888975	-3.937275	0.000000
C	13.974764	-5.735151	0.000000
H	14.500169	-6.707895	0.000000
C	14.801336	-4.554917	0.000000
C	16.140561	-4.707172	0.000000
H	16.596661	-5.697574	0.000000
O	0.519651	-1.081778	0.000000
O	5.189157	-1.742557	0.000000
H	5.752489	-2.562889	0.000000
C	1.567436	-1.734622	0.000000
H	1.484382	-2.838167	0.000000
C	2.895152	-1.207257	0.000000
C	3.925395	-2.093054	0.000000
H	3.727777	-3.167976	0.000000
O	-5.360675	1.361311	0.000000
O	-0.643371	1.252178	0.000000
H	0.000000	0.499271	0.000000
C	-4.242505	0.838668	0.000000
H	-4.191239	-0.266854	0.000000
C	-2.986077	1.519955	0.000000
C	-1.860254	0.759668	0.000000
H	-1.929531	-0.330533	0.000000
O	-11.444301	3.396632	0.000000
O	-6.730347	3.588713	0.000000
H	-6.035031	2.884487	0.000000
C	-10.300690	2.935535	0.000000
H	-10.185099	1.834483	0.000000
C	-9.085219	3.693061	0.000000
C	-7.911896	3.011817	0.000000
H	-7.905015	1.919448	0.000000
O	-16.985545	7.021904	0.000000
O	-12.403879	5.868891	0.000000
H	-11.923733	5.005952	0.000000
C	-16.044846	6.243630	0.000000
H	-16.240050	5.146646	0.000000
C	-14.643954	6.613797	0.000000
C	-13.709571	5.639710	0.000000
H	-14.005973	4.587965	0.000000
H	-14.372731	7.664427	0.000000
H	-9.115875	4.777546	0.000000
H	-2.944458	2.603834	0.000000
H	3.064982	-0.135974	0.000000
H	8.579439	-2.662860	0.000000
H	14.344822	-3.570526	0.000000
C	-1.435003	13.314693	0.000000
C	-0.378040	12.221768	0.000000
H	0.267237	12.331001	0.886397
H	-2.085322	13.214991	0.876955
H	-0.484141	7.297445	0.876482
H	-0.661554	0.405290	0.876929
H	-0.459256	-6.416019	0.876821
H	-1.185866	-12.555913	0.876638
H	-2.085322	13.214991	-0.876955
H	0.267237	12.331001	-0.886397
H	-0.484141	7.297445	-0.876482
H	1.173196	5.403341	-0.886267
H	-0.661554	0.405290	-0.876929
H	1.105503	-1.386592	-0.886219
H	-0.459256	-6.416019	-0.876821
H	1.351813	-8.161729	0.886982
H	-1.185866	-12.555913	-0.876638
H	-0.316852	-14.912835	-0.889109

6 MOLECULES

O	7.767283	-1.724445	0.000000
O	12.090704	0.279603	0.000001
H	13.030713	0.042738	0.000001
C	8.981124	-1.766182	0.000000
H	9.480250	-2.757018	0.000000
C	9.870758	-0.556055	0.000000
C	11.349287	-0.914761	0.000001
H	11.580450	-1.525947	0.886779
O	14.736977	-0.807906	0.000001
O	17.596561	2.986590	0.000001
H	18.514888	3.268505	0.000001
C	15.835580	-0.291505	0.000001
H	16.732906	-0.943292	0.000001
C	16.062559	1.194428	0.000001
C	17.534663	1.576414	0.000001
H	18.029199	1.157568	0.889444
O	0.941585	-0.811564	0.000000
O	5.610478	0.127851	0.000000
H	6.469545	-0.320145	0.000000
C	2.111583	-1.135329	0.000000
H	2.367047	-2.215664	0.000000
C	3.260477	-0.168207	0.000000
C	4.614217	-0.863174	0.000000
H	4.696870	-1.511652	0.886585
O	-5.714574	-0.603255	0.000000
O	-1.237714	1.037019	0.000000
H	-0.328933	0.705987	0.000000
C	-4.507913	-0.736430	0.000000
H	-4.085701	-1.762981	0.000000
C	-3.524533	0.398831	0.000000
C	-2.083258	-0.086565	0.000000
H	-1.904329	-0.716210	0.885346
O	-12.447338	-1.115514	-0.000001
O	-8.128868	0.895930	0.000000
H	-7.191850	0.654475	0.000000
C	-11.233264	-1.155532	-0.000001
H	-10.731507	-2.145365	-0.000001
C	-10.346595	0.056666	0.000000

	C -8.868014 -0.299775 0.000000
	H -8.634098 -0.910924 0.885880
	O -19.079606 1.021249 -0.000001
	O -14.308667 1.052589 -0.000001
	H -13.556263 0.441657 -0.000001
	O -17.999005 0.476990 -0.000001
	H -17.944659 -0.634965 -0.000001
	C -16.680635 1.210382 -0.000001
	C -15.482064 0.274434 -0.000001
	H -15.522534 -0.378671 0.886367
	H -16.651270 1.867537 -0.877279
	H -10.594606 0.667754 -0.876403
	H -3.720164 1.027671 -0.876878
	H 3.165518 0.483663 -0.876896
	H 9.624594 0.055264 -0.876626
	H 15.559970 1.620509 -0.876523
	H -16.651270 1.867537 0.877278
	H -15.522534 -0.378671 -0.886369
	H -10.594606 0.667754 0.876402
	H -8.634098 -0.910924 -0.885881
	H -3.720164 1.027671 0.876878
	H -1.904329 -0.716210 -0.885346
	H 3.165518 0.483663 0.876897
	H 4.696870 -1.511652 -0.886584
	H 9.624594 0.055264 0.876627
	H 11.580450 -1.525947 -0.886778
	H 15.559970 1.620509 0.876525
	H 18.029199 1.157568 -0.889443
